# Set-Up of Bacterial Cellulose Production From the Genus *Komagataeibacter* and Its Use in a Gluten-Free Bakery Product as a Case Study

**DOI:** 10.3389/fmicb.2019.01953

**Published:** 2019-09-06

**Authors:** Ileana Vigentini, Vincenzo Fabrizio, Federico Dellacà, Sergio Rossi, Isabella Azario, Cristiano Mondin, Maurizio Benaglia, Roberto Foschino

**Affiliations:** ^1^Department of Food, Environmental and Nutritional Sciences, University of Milan, Milan, Italy; ^2^Biotechnology Division, LaVitaWiz, Wiz Chemicals, Dairago, Italy; ^3^Department of Chemistry, University of Milan, Milan, Italy; ^4^Farine Varvello & Co., Milan, Italy

**Keywords:** bacterial cellulose, *Komagataeibacter* (*Gluconacetobacter*), *Komagataeibacter rhaeticus*, gluten-free products, bread additives

## Abstract

The use of bacterial cellulose (BC) in food systems is still limited due to production costs. Nine clones belonging to *Komagataeibacter hansenii*, *Komagataeibacter nataicola*, *Komagataeibacter rhaeticus*, *Komagataeibacter swingsii*, and *Komagataeibacter xylinus* species were screened for cellulose productivity in growth tests with five different carbon sources and three nitrogen sources. The water-holding and rehydration capacities of the purified cellulose were determined. The structure of the polymer was investigated through nuclear magnetic resonance (NMR) spectroscopy, attenuated total reflection Fourier transform infrared (ATR-FT-IR) spectroscopy and X-ray diffraction (XRD) analysis, and observed by scanning electron microscope (SEM). Natural mutants of *K. rhaeticus* LMG 22126^T^ and *K. swingsii* LMG 22125^T^ showed different productivity. The factors “bacterial isolate” and “nitrogen source” significantly affected the production of cellulose (*p* < 0.01) rather than the factor “carbon source” (*p* = 0.15). However, on average, the best conditions for increasing yield were found in medium containing glucose and peptone. Water-holding capacity (WHC) values ranged from 10.7 to 42.3 (*g*_water_/*g*_cellulose_) with significant differences among strains (*p* < 0.01), while the rehydration capacity varied from 4.2 to 9.3 (*g*_water_/*g*_cellulose_). A high crystallinity (64–80%) was detected in all samples with Iα fractions corresponding to 67–93%. The ATR-FT-IR spectra and the XRD patterns confirmed the expected structure. BC made by GVP isolate of *K. rhaeticus* LMG 22126^T^, which was the strain with the highest yield, was added to a gluten-free bread formulation. Results obtained from measurements of technological parameters in dough leavening and baking trials were promising for implementation in potential novel foods.

## Introduction

In food technology, cellulose and its derivatives obtained after partial etherification with different groups, are approved additives coded from E460 to E469 (Commission Regulation, 2012), used for their thickening, anti-caking, gelling, and stabilizing properties. In the case of gluten-free bakery products, cellulose and its derivatives are valuable ingredients to provide the required viscoelasticity of the dough since the protein net of glutenins and gliadins is missing (Rai et al., 2018). Microcrystalline cellulose is currently obtained from wood pulp of different plants through mechanical and chemical processes, which can entail a negative ecological impact in terms of wasteful consumption of trees, water depletion, energy usage and environmental pollution (Moon et al., 2016; Sun et al., 2018).

The interest in bacterial cellulose (BC) is increasing in several manufacturing sectors (Augimeri et al., 2015; Gallegos et al., 2016; Gullo et al., 2018) as this homopolysaccharide is produced by various microorganisms in a form with high purity, free from lignin and hemicelluloses, with high crystallinity, water adsorption and tensile strength, allowing innovative applications which have never been previously exploited (Azeredo et al., 2019). Cellulose generated by the genus *Komagataeibacter* (Yamada et al., 2012), formerly *Gluconacetobacter*, can be an eligible alternative for the food industry and it may be recognized as harmless because its chemical structure is well known and identical to that of the vegetable one. Acetic acid bacteria that synthesize it belong to the risk group 1 according to the German TRBA classification (Federal Institute for Occupational Safety and Health, 2010). However, these microorganisms and their extracellular products are naturally present in various traditional foods with a long history of safety for human consumption, like kombucha (Gomes et al., 2018; Villarreal-Soto et al., 2018), nata (Dourado et al., 2017; Zhang et al., 2017), water kefir (Gulitz et al., 2011), lambic beer (De Roos and De Vuyst, 2018), and numerous kinds of vinegars and liquors (Gullo and Giudici, 2008; Štornik et al., 2016). Bourdichon et al. (2012) presented an inventory of microbial cultures used in food fermentations in which *Gluconacetobacter* was involved. Recently, the EFSA Panel on Biological Hazards (BIOHAZ) included the species *Komagataeibacter sucrofermentans* in the list QPS-recommended biological agents intentionally added to food (European Food Safety Authority, 2019), opening the way toward the acceptance of BC, although it is only for production purposes. In 2017, Dourado et al. (2017) revised the toxicological and dietetic aspects of BC in an animal model, concluding that BC from *Komagataeibacter* is safe for applications in food technology. Volova et al. (2018) demonstrated that native BC did not cause cytotoxicity upon contact with mouse fibroblast cells. In an earlier study, Chau et al. (2008) interestingly reported that the BC from *Acetobacter xylinum* showed a significantly higher effect in lowering serum lipids and cholesterol than plant cellulose in hamsters. In 2010, Jagannath et al. (2010) proposed the use of BC from the same bacterial species as a cryoprotective and carrier to support probiotic bacteria in starter cultures production and dietary administration. As the literature makes clear, the amount and the quality of BC produced, as well as its mechanical properties, are strain dependent traits and they may change on the basis of the carbon sources consumed (Zhong et al., 2013; Fang and Catchmark, 2015; Valera et al., 2015; Molina-Ramírez et al., 2017; Chen et al., 2018; Wang et al., 2018). In addition, pH, temperature and protocols of cultivation are environmental factors affecting BC production (Thorat and Dastager, 2018; Volova et al., 2018). A higher yield is normally achieved by culturing bacterial cells in static conditions rather than with agitation, since in the latter case the development of mutant cells that reduce the polysaccharide synthesis is promoted (Krystynowicz et al., 2002; Volova et al., 2018). Stationary cultures normally produce cellulose in gelatinous films consisting of a nanofibrillary network, whereas shaking ones lead to the appearance of irregularly shaped sphere-like particles with a lesser tight structure (Gullo et al., 2018; Singhsa et al., 2018). Since BC productivity is generally low for making it an economically acceptable process for industrial implementation, selection for better performing strains and assessment of the most efficient conditions for cellulose synthesis are still under study (Campano et al., 2016; Chen et al., 2018).

In the present work, we examined the effect of five different carbon and three nitrogen sources for cellulose synthesis by various *Komagataeibacter* strains in order to increase production and reduce the costs for scaling up in food applications. We evaluated the difference in genotype between high cellulose producing cells and low cellulose producing mutants. BC productivity, water-holding capacity (WHC) and rehydration capacity were determined and yield was improved for two strains by setting up a protocol. To investigate the structure of the purified polymers, cellulose samples were characterized by nuclear magnetic resonance (NMR) spectroscopy (^13^C CP/MAS NMR, ^1^H and ^13^C HRMAS), attenuated total reflection Fourier transform infrared spectroscopy (ATR-FT-IR) and X-ray diffraction (XRD). BC from *Komagataeibacter* species can be usefully applied in food preparations for various purposes just as well as the cellulose from vegetable origin. We introduced it in a gluten-free formulation for baked goods as a structuring agent in place of the gluten network of native wheat for improving the material of the loaves in sensory features and shelf life duration. From a nutritional point of view, BC can be considered a promising low-calorie fiber-rich ingredient or fat substitute, in compliance with current legislation that regulates the requirements for novel food introductions.

## Materials and Methods

### Bacterial Strains and Growth Conditions

In this study, *Komagataeibacter hansenii* LMG 1527^T^, *Komagataeibacter nataicola* LMG 1536^T^, *Komagataeibacter rhaeticus* LMG 22126^T^, *Komagataeibacter swingsii* LMG 22125^T^, *Komagataeibacter xylinus* LMG 1515^T^, and LMG 1518 strains deriving from BCCML/LMG Bacteria Collection were investigated for the ability to produce cellulose. Each strain was streaked two consecutive times on ACE medium (Dellaglio et al., 2005) and cell suspensions from colonies were observed by optical microscope (Axio Lab. A1 model, Zeiss, Oberkochen, Germany). The plates were incubated at 28°C for 5 days in aerobic conditions. The purified isolates were stored at −80°C in ACE broth with added 25% (v/v) glycerol. Fresh cells for the inoculum preparation in growth tests were cultivated in 25 mL of ACE broth at 28°C for 5 days at 120 rpm by seeding 1% (v/v) of thawed stock cultures.

### PFGE Typing Analysis

DNAs of the bacterial isolates were subjected to Restriction Fragment Length Polymorphism analysis in order to evaluate the intra-specific diversity and recognize gross alterations in bacterial chromosome. After incubation, fresh cells were treated with 5 mg/mL of cellulase from *Trichoderma longibrachiatum* (Sigma-Aldrich, St. Louis, MO, United States) at 37°C for 24 h in order to degrade cellulose and reach an optical density value of approximately 1.0 at 600 nm (UV–visible spectrophotometer, Jenway model 7315, Bibby Scientific, Stone, United Kingdom). The protocol for setting up the assay was inspired by Picozzi et al. (2010). DNA digestion with *Xba*I endonuclease (Fermentas, Thermo Fischer Scientific, Waltham, MA, United States) was performed overnight at 37°C in a 100 μL total volume of the specific buffer with 5U of restriction enzyme. DNA fragments were separated in 1% (w/v) Pulsed Field Certificate Agarose (Bio-Rad, Hercules, CA, United States) in 0.5 X TBE (45 mM Tris, 45 mM boric acid, 1 mM EDTA, pH 8.0) with CHEF-DR II Apparatus (Bio-Rad). Electrophoresis was performed at 14°C at a constant voltage of 4.5 V/cm with a switch time ramped from 5 to 45 s for 24 h. Gels were stained with ethidium bromide (0.5 μg/mL) and photographed under UV light by Gel doc XR (Bio-Rad).

### Strain Selection for Cellulose Production by Carbon and Nitrogen Sources

Cell growth and cellulose production of each isolate were evaluated by comparing the utilization of different carbon and nitrogen sources in a basal medium (yeast extract 5 g/L, pH 6.5). The test was carried out in six-well polystyrene microplates and the optical density of the culture at 600 nm was monitored by a Plate reader (Infinite 200pro, Tecan Group, Zurich, Switzerland). Cells were grown in 8 mL broth with five different carbon sources (fructose, glucose, glycerol, maltose, and sucrose) in equimolar amounts (0.25M) combined one by one with three different nitrogen sources (ammonium sulfate, peptone and urea) in equimolar amounts (0.04M or 5 g/L for peptone). Master solutions of the carbon and nitrogen sources were separately sterilized by filtration (0.22 μm) and diluted to the basal medium at the needed concentrations. All isolates were inoculated at 0.10 OD_600__nm_ (approximately 4 × 10^6^ UFC/ml) and microplates were incubated at 28°C for 5 days in static conditions. At the end of the test the pH of the medium was measured by a pH meter (3510 model, Jenway) equipped with an appropriate probe for small volume liquid samples (924 904 type, Jenway). The optical density of the bacterial cultures was determined after removing the gels formed. Samples were transferred to glass beakers and treated with 0.5M NaOH at 90°C for 15 min to eliminate cells and residual materials. Cellulose samples were then harvested on pre-weighed and dried paper filters (Whatman grade 4, Sigma-Aldrich), thoroughly washed with distilled water and subjected to vacuum pumping through a Büchner funnel to eliminate the excess liquid. Filters were placed on glass Petri dishes and dried in a hot air oven at 105°C until invariable weight. The amount of cellulose was calculated as the difference between the weight of the paper filter with the dried gel and the preceding weight without it. Each test was replicated at least three times.

### Cellulose Water-Holding Capacity, Rehydration Capacity, and Yield Optimization

The experiments were carried out in 500 mL Erlenmeyer flasks with 175 mL of sterile broth containing glucose (45 g/L), peptone (5 g/L), yeast extract (5 g/L), pH 6.5. Fresh cells were prepared as before reported, inoculated at 0.10 OD_600__nm_ and flasks were incubated at 28°C for 5 days in static conditions. The formed films or flakes were purified by the abovementioned alkaline treatment, thoroughly washed and filtered as described in previous section. BC samples were removed from the paper filters and weighed on glass Petri dishes. They were dried in a hot air oven at 105°C until invariable weight and weighed again. The WHC was calculated as the difference between the weights of the cellulose film before and after the drying treatment, divided by the weight of the dried film. Dried BC samples were rehydrated in glass Petri dishes by soaking in distilled water at 25°C for 30 min. The gels were put on paper filters and treated as described above to eliminate the excess liquid. BC samples were then removed from the paper filters and weighed on glass Petri dishes. The rehydration capacity was calculated as the difference between the weights of the wet rehydrated film and the dried film, divided by the weight of the dried film. To optimize the yield of cellulose production some trials were performed for a few selected strains. In particular, after the first cultivation at 28°C for 5 days in static conditions, the floating films on the liquid surface were removed and the flasks with the remaining medium were incubated twice again at 28°C for 5 days, by taking out the hydrated gels each time. The pH value of the medium was recorded as previously described. The samples were treated and dried as already reported in the preceding section in order to determine the BC weights. The carbon conversion rates were calculated as percent ratio between the dry weight of BC (g) produced by a strain and the weight of carbon source (g) put in the cultural medium (Chen et al., 2017). Three replicates were carried out for each experiment.

### NMR Spectroscopy

The structure of BC samples deriving from experiments performed as described in the previous section was investigated by applying two NMR spectroscopy techniques. Before the measurement, cellulose films were lyophilized for 24 h using Telstar Cryodos Freeze Dryer (Terrassa, Spain). Solid state ^13^C CP/MAS spectra were analyzed and recorded with a Brüker Avance 500 spectrometer (Brüker, Billerica, MA, United States) equipped with a 4 mm Magic Angle Spinning (MAS) broad-band probe, at a temperature of 300°K and 7 kHz of spinning and at 125.62 MHz. Approximately 0.1 g of sample were packed into a 4 mm MAS rotor. ^13^C nuclei were observed using the cross-polarization (CP) methods, according to the protocols of Castro et al. (2011) and Idström et al. (2016). All chemical shifts were externally referenced to tetramethylsilane (TMS). No resolution improvement was found at higher spinning rate and/or temperature.

^1^H and ^13^C HRMAS (High Resolution Magic Angle Spinning) NMR were performed with the same spectrometer operating a 500 and 125.62 MHz, respectively, at room temperature, following the procedure of Rueda et al. (2003). The compounds were allowed to swell in D_2_O, with TMS as internal reference. ^13^C {1H} spectra were obtained using Waltz decoupling and were exponentially multiplied to give 0.8 Hz line broadening before Fourier transformation, using standard Brüker software sequences.

### Attenuated Total Reflection Fourier Transform Infrared Spectroscopy

Bacterial cellulose samples were lyophilized before analyzing as already described. The ATR-FT-IR spectra were acquired and recorded on a Perkin Elmer Spectrum BX II FT-IR System (Waltham, MA, United States) in the 4000–600 cm^–1^ range with a resolution of 4 cm^–1^ and an accumulation of 16 scans. Spectra analysis was carried out using the software provided with the instrument.

### Scanning Electron Microscope

Scanning electron microscope (SEM) images were obtained from two lyophilized BC samples and examined using a JEOL JSM 5500 LV scanning microscope (JEOL, Tokyo, Japan) equipped with backscattered electrons detector (BSE).

### X-Ray Diffractometric Analysis

X-ray diffraction patterns were obtained using a Brüker D8 powder diffractometer (Brüker) with Cu-Kα radiation, operating at 40 kV and 40 mA. The crystallinity index (CI) was calculated according to the protocol of Park et al. (2010): % CI 1/4XA 100; 010; 110 = XA 100; 010; 110; Am where A100, A010, A110, and Aam are the areas under the curve of the 100, 010, 110, and amorphous peaks, respectively.

### Bread-Making Test With Gluten-Free Formulation Added With BC

The recipe of the mixture, excluding water and wet BC, was composed by rice flour 48.8% (w/w), corn starch 21.2%, quinoa flour 17.1%, glucose 3.4%, rice oil 2.8%, fresh compressed yeast 1.8%, xanthan gum 1.7%, guar gum powder 1.7%, salt 1.6%. Hydrated gels derived from fresh cultures grown according to the protocol for Cellulose Water-Holding Capacity but in larger volumes, were thoroughly washed with distilled water, minced by means of a blender (Koenic KHB 610, Media-Saturn Holding, Ingolstadt, Germany) and added to the dough mixture in such a way as to represent 1.25% (w/w) of the final formulation, expressed as dry cellulose. Samples without the BC addition were kneaded and baked, as negative control. Other dough and bread samples were made using a gluten-free flour mix, as positive control, purchased on the Italian market. In this formulation, the cellulose was present as hydroxyl-propyl-methyl cellulose (HPMC, food additive number E464) representing 2.0% (w/w) of the dry weight. Water absorption and dough consistency were assessed with a Brabender^®^ Farinograph (Brabender OHG, Duisburg, Germany; at 30°C). The doughs were produced in a planetary mixer (Ostali power speed, Ing. Polin E C. S.p.A., Verona, Italy) through a mixing step of dry ingredients, addition of oil, yeast, water and minced BC lasting 5 min, followed by a kneading step of 15 min. The resulting doughs were divided into aliquots, 500 g each, placed into baking molds and leavened in a climatic chamber at 30°C and 75% of relative humidity for 3 h. Dough development during leavening, gas production and retention, were investigated in by a rheofermentometer (Rheo F4 Chopin; Chopin Technologies, Villeneuve-la-Garenne, France) by controlling Hm (dough maximum height; mm), Hf (dough height at the end of the test; mm), Tx (time of dough porosity appearance; min), total, released and retained CO_2_ volumes, and Rc (gas retention coefficient; %). Microbiological counts were carried out at the end of fermentation time to control yeast and acetic acid bacteria populations in dough samples by cultural techniques (Gulitz et al., 2011). The concentrations of acetic and gluconic acids were determined by specific enzymatic kits based on a spectrophotometric protocol according to the supplier’s recommendations (Megazyme International, Bray, Ireland). The leavened doughs were then baked in an electric oven (Polin, Ing. Polin E C. S.p.A., Verona, Italy) at 250°C for 35 min, then cooled at room temperature for 1 h. After removing from the molds, bread loaves were sliced (20 mm thickness) and characterized for texture and crumb porosity. Crumb firmness was assessed by means of a compression test performed with a TA-XT Texture Analyzer (Stable Micro Systems, Surrey, United Kingdom), equipped with a 500 N load cell. Samples were compressed up to 40% deformation, using a 36 mm diameter cylindrical probe, at a compression speed of 1.7 mm/s. The following parameters were evaluated: crumb hardness (N; load at 25% deformation) Three replicates were analyzed for each recipe, at initial time and after 24 h of storage at room temperature. Bread crumb porosity was evaluated by image analysis with an optical scanner (Epson Perfection 2480 Photo, Seiko Epson Corporation, Suwa, Japan).

### Statistical Analysis

The IBM SPSS statistics 25 software was used for statistical analysis and graphs drawing. One-way analysis of variance (ANOVA) was carried out to investigate the differences between the obtained mean values for any factor. Discrimination was based on Tukey’s multiple comparisons test.

## Results

### Observation and Identification of Different Colony Morphotypes

The type strains of five *Komagataeibacter* species, which are considered the highest producers of cellulose, and other isolates were selected. Despite consecutive steps of purification, different colony shapes were observed for two strains in ACE agar medium. In particular, *K. rhaeticus* LMG 22126^T^ strain showed small and rough colonies of about 0.5 mm in diameter (bacterial isolates named GDP and GVP), as predominant morphotypes in the culture, and large and smooth colonies of about 1.5 mm of diameter (isolate named GDG) with lower occurrence. In the same way, *K. swingsii* LMG 22125^T^ strain more frequently formed small and rough colonies of about 0.5 mm of diameter (isolate named GSP) and large and smooth colonies of about 1.0 mm of diameter (isolate named GSG), as a minority type. Microscopic observations of colony samples showed regular rods in short chains without differences between the two forms. The RFLP analysis on DNA samples, which were obtained from colonies with diverse shapes revealed undistinguishable restriction patterns (Supplementary Figure S1), thus indicating that isolates were different clones of the same strain. This evidence led us to think that colony shape was not related to genomic rearrangement but probably relied on a distinctive genic expression affected by environmental conditions. However, the isolates with different phenotypes coming from the same strain were separately considered for the following analyses.

### Effect of Different Carbon and Nitrogen Sources on Cell Growth and Cellulose Production

The ability to make cellulose of nine isolates deriving from six *Komagataeibacter* sp. strains was tested in a basal medium by combining five different carbon sources (fructose, glucose, glycerol, maltose, and sucrose) with three nitrogen sources (ammonium, peptone and urea). Mean values ± standard deviations of optical density, final pH in growth medium and BC production are reported in Supplementary Table S1. The reported data of OD_600__nm_ are the difference between the final and the initial value. The determination of optical density at the end of the incubation time was performed after having taken out the formed hydrated gels. While most clones made an easily removable pellicle, isolate GDG of *K. rhaeticus* LMG 22126^T^, isolate GSG of *K. swingsii* LMG 22125^T^ and *K. xylinus* LMG 1515^T^ strain produced small flakes of cellulose. This made it difficult to recover the polysaccharide matrix and to determine the optical density only resulting from the bacterial cells. However, even though cells might be mostly embedded in a BC structure, the effect of the carbon source on cell growth was considerable. Regardless of the nitrogen source used, the mean OD_600__nm_ values of the set of isolates grown in media with glucose (mean value = 0.37 ± standard deviation 0.24) were significantly higher (*p* < 0.01) than those found in the media with glycerol (0.29 ± 0.24), maltose (0.27 ± 0.16) or sucrose (0.27 ± 0.19). On the other hand, the nitrogen source did not significantly affect the final cell concentration (*p* = 0.23), though higher values were attained in the presence of ammonium sulfate (OD_600__nm_ = 0.37 ± 0.17) rather than with peptone or urea (0.30 ± 0.32 and 0.29 ± 0.16, respectively). In any case, the optical densities of the samples were not significantly correlated with the polymer production, since the linear regression analysis revealed a low value of the determination coefficient (R^2^ = 0.064) between these two quantities.

The acidification levels measured in the media at the end of incubation was consistent to the data of optical densities since the mean values of pH in growth tests with glucose (mean value = 3.31 ± standard deviation 0.83) were significantly lower (*p* < 0.01) than those with glycerol, maltose or sucrose (5.08 ± 1.44, 5.10 ± 1.44, and 5.19 ± 1.61, respectively). Therefore, the achievement of a higher cell concentration was associated with an increasing acidity in the cultural broth, most likely due to a higher carbon consumption and related to the type of carbohydrate consumed. As regards the effect of the nitrogen source, the greatest lowering of pH, independently of the used carbon source, was detected in trials with ammonium sulfate (3.63 ± 0.86) in comparison to those with urea (4.91 ± 1.62) or with peptone (5.59 ± 1.21) (*p* < 0.01). This last ingredient had probably exerted a stronger buffer action in the growth medium.

**FIGURE 1:**
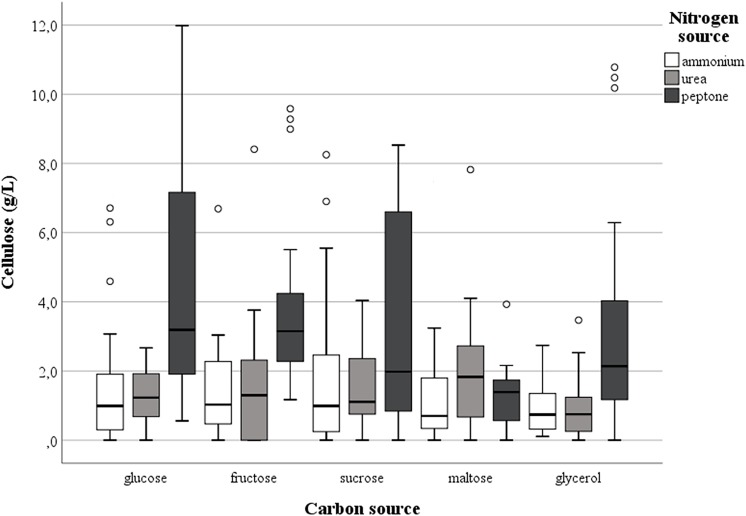
Effect of carbon and nitrogen sources on the cellulose production by the set of strains investigated.

**FIGURE 2:**
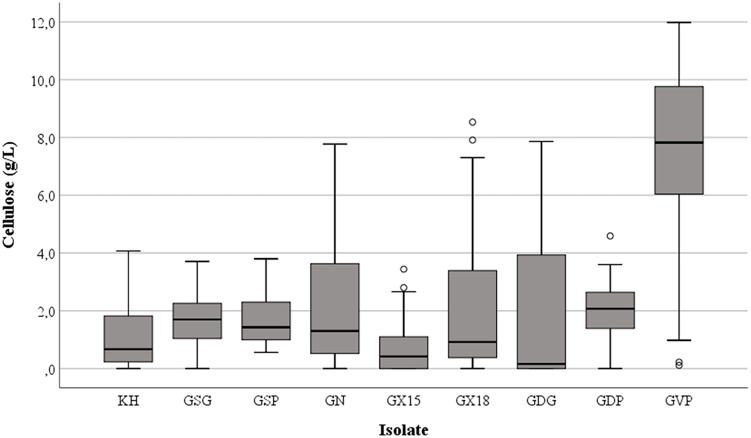
Cellulose production by different bacterial isolates. KH = *K. hansenii* LMG 1527^T^ strain; GSG and GSP isolates of *K. swingsii* LMG 22125^T^ strain; GN = *K. nataicola* LMG 1536^T^ strain; GX15 = *K. xylinus* LMG 1515^T^ strain; GX18 = *K. xylinus* LMG 1518 strain; GDG, GDP and GVP isolates of *K. rhaeticus* LMG 22126^T^ strain.

Cellulose production was observed in most of the tested conditions, even if the amounts were different as functions of medium composition and the isolates investigated. Regardless of the nitrogen source, the highest harvest of cellulose was obtained with glucose (mean value = 2.85 g/L ± standard deviation 2.61) while the lowest was with glycerol (1.74 g/L ± 2.23), with intermediate values for fructose (2.45 g/L ± 2.38), sucrose (2.52 g/L ± 2.48) or maltose (1.98 g/L ± 2.57); however, the differences were not significant (*p* = 0.15). On the other hand, the presence of peptone in the growth medium considerably enhanced (*p* < 0.01) the cellulose production to a mean value of 3.40 g/L ± 3.07 in comparison with 1.83 g/L ± 2.10 or 1.73 g/L ± 2.15 found with ammonium sulfate and urea, respectively. In particular, strain *K. hansenii* LMG 1527^T^ generated negligible quantities of cellulose when ammonium sulfate was the only nitrogen source, while isolate GDG of *K. rhaeticus* LMG 22126^T^ strain did not make a quantifiable polymer amount with urea in the medium. Figure 1 shows the spread of cellulose production and the relevant median values in the strains examined, as a function of the carbon and nitrogen sources used. Not considering the single tested isolates, the best combinations occurred when glucose, fructose or sucrose were used with peptone. Furthermore, the effect of the single isolate on the polymer synthesis proved to be significant (*p* < 0.01). As displayed in Figure 2, the clone named GVP of *K. rhaeticus* LMG 22126^T^, independently of carbon and nitrogen sources, was the highest cellulose producer with an overall average of 7.23 g/L ± 3.25. It was followed by isolates GN of *K. nataicola* LMG 1536^T^ (2.27 g/L ± 2.36) and GDP of *K. rhaeticus* LMG 22126^T^ (2.04 g/L ± 0.89), whereas GX15 of *K. xylinus* LMG 1515^T^, KH of *K. hansenii* LMG 1527^T^ and GSG of *K. swingsii* LMG 22125^T^, were significantly the lowest ones with mean values of cellulose production of 0.75 g/L ± 0.85, 1.13 g/L ± 1.04, and 1.57 g/L ± 1.00, respectively. It is worth noting that clones from the same strain of *K. rhaeticus* LMG 22126^T^ (GDG and GVP) revealed a significant diversity in cellulose production (*p* < 0.01), meanwhile the different colony phenotypes have already been mentioned in a previous section.

### Determination of Bacterial Cellulose Characteristics and NMR Analysis Results

Taking into account the previous results, BC produced by the tested isolates were compared through cultivation experiments carried out in 175 mL volumes of medium containing glucose (45 g/L) and peptone (5 g/L). The bacterial cultures were incubated at 28°C for 5 days in static conditions. The WHC of the cellulose samples proved to be significantly different (*p* < 0.01) among the strains investigated (Table 1). WHC values ranged from 10.7 ± 1.1 of *K. xylinus* LMG 1515^T^ to 42.3 ± 2.2 (*g*_water_/*g*_cellulose_) of the isolate GSG from *K. swingsii* LMG 22125^T^. This last strain synthesized a type of polymer that retained more water than the others did. Interestingly, clones deriving from the same strain, like GDG, GDP, GVP of *K. rhaeticus* LMG 22126^T^ or GSG and GSP of *K. swingsii* LMG 22125^T^, showed the same WHC despite having produced different quantities of cellulose. The rehydration capacity of dried films ranged from 4.2 *g*_water_/*g*_cellulose_ of isolate GSG of *K. swingsii* LMG 22125^T^ to 9.3 *g*_water_/*g*_cellulose_ of *K. hansenii* LMG 1527^T^, without significant differences (*p* = 0.51) (Table 1). For each tested strain, the aptitude to absorb water after drying was not linked to the WHC found earlier.

**TABLE 1:** Characteristics of BC samples obtained by different strains or isolates grown in static conditions at 28°C for 5 days.

		**Water holding capacity**	**Rehydration capacity**	**Crystallinity (%)**	**Iα fraction (%)**
**Strain**	**Isolate**	**(*g*_water_/*g*_cellulose_)**	**(*g*_water_/*g*_cellulose_)**	**± 5**	**± 2**
*K. hansenii* LMG 1527^T^		25.9^a,b^ ± 5.8	9.3 ± 4.3	75	84
*K. nataicola* LMG 1536^T^		22.5^a,b^ ± 9.1	7.4 ± 0.2	80	89
*K. rhaeticus* LMG 22126^T^	GDG	18.1^a^ ± 7.9	8.5 ± 4.3	72	88
	GDP	15.7^a^ ± 1.0	5.0 ± 3.3	73	90
	GVP	18.7^a^ ± 4.5	4.3 ± 1.9	72	84
*K. swingsii* LMG 22125^T^	GSG	42.3^c^ ± 2.2	4.2 ± 2.5	65	93
	GSP	34.5^b,c^ ± 5.9	5.5 ± 3.3	64	67
*K. xylinus* LMG 1515^T^		10.7^a^ ± 1.1	8.0 ± 2.7	77	86
*K. xylinus* LMG 1518		20.7^a,b^ ± 3.5	8.5 ± 1.6	74	84

*Water holding capacity and rehydration capacity values are mean of three replicates ± standard deviation. Values with different superscripts of lowercase letter in the same column for the each bacterial strain or isolate are significantly different (*p* < 0.01).*

**FIGURE 3:**
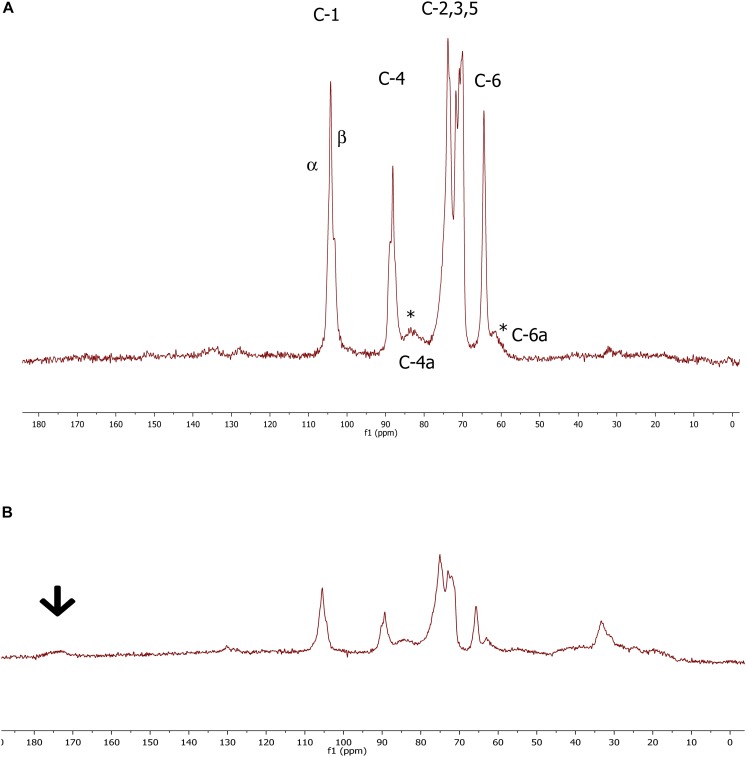
^13^C CP/MAS spectrum of cellulose samples from GSP **(A)** and GSG isolate **(B)** derived from *K. swingsii* LMG 22125^T^ strain. Chemical shifts are shown in parts per million, relative to Me_4_Si. The arrow shows the resonance of the carboxylic carbon. α and β symbols refer to the criystalline forms of BC. ^*^Highlights the presence at 83.0 and 62.1 ppm of C4 and C6 signals related to the amorphous form of cellulose samples derived from GSP isolate.

The ^13^C CP/MAS NMR spectroscopy was used to examine the molecular properties of the BC samples formed by the different isolates. In preliminary tests, raw materials showed some traces of impurities, displaying broad signals between 20 and 60 ppm in the aliphatic region (data not shown); however, after the purification protocol, all by-products were removed. In general, cellulose films made by the investigated strains revealed highly comparable spectra allowing us to estimate the purity of each specimen and the related crystallinity percentage (Table 1), which varied from 64 to 80%. The broad signals detected in all spectra were related to the amorphous cellulose. Moreover, isolates GDG, GDP, and GVP of *K. rhaeticus* LMG 22126^T^ strain or isolates GSG and GSP of *K. swingsii* LMG 22125^T^ strain had very similar values for crystallinity. Meaningful signals in the peaks of the crystalline form were those corresponding to C-1 and C-4 carbons, which exhibited the characteristic splitting, providing the evidence of α and β glycoside linkages. The splitting of C-1 signals at 104.0 and 103.5 ppm was ascribed to the Iα and Iβ forms, always present with values ranging from 3:2 to 4:1 ratios. Iα fraction ranged from 67 to 93%. Spectra of cellulose samples from isolates GSP and GSG of *K. swingsii* LMG 22125^T^ are shown in Figure 3. The resonances at 89.1 and 64.0 ppm, shown as C-4 and C-6, respectively, were ascribed to the crystalline form, whereas the broad signals at 83.0 and 62.1 ppm (Figure 3A) indicated with asterisks, were related to C-4 and C-6 of the amorphous region, respectively. Remarkably, even after the purification step, the sample of cellulose from isolate GSG of *K. swingsii* LMG 22125^T^ (Figure 3B) and strain *K. nataicola* LMG 1536^T^ (data not shown) presented significant traces of esters and/or acid derivatives, easily recognized from the broad resonance a 172 ppm. Since the product from isolate GSP of *K. swingsii* LMG 22125^T^ proved to be soluble in deuterated water, the ^1^H and ^13^C NMR spectra were also carried out in solution (Supplementary Figure S2). Results confirmed that the cellulose solubility decreases as the amorphous form increases in the composition of the polymer.

### Results of ATR-FT-IR Analysis

**FIGURE 4:**
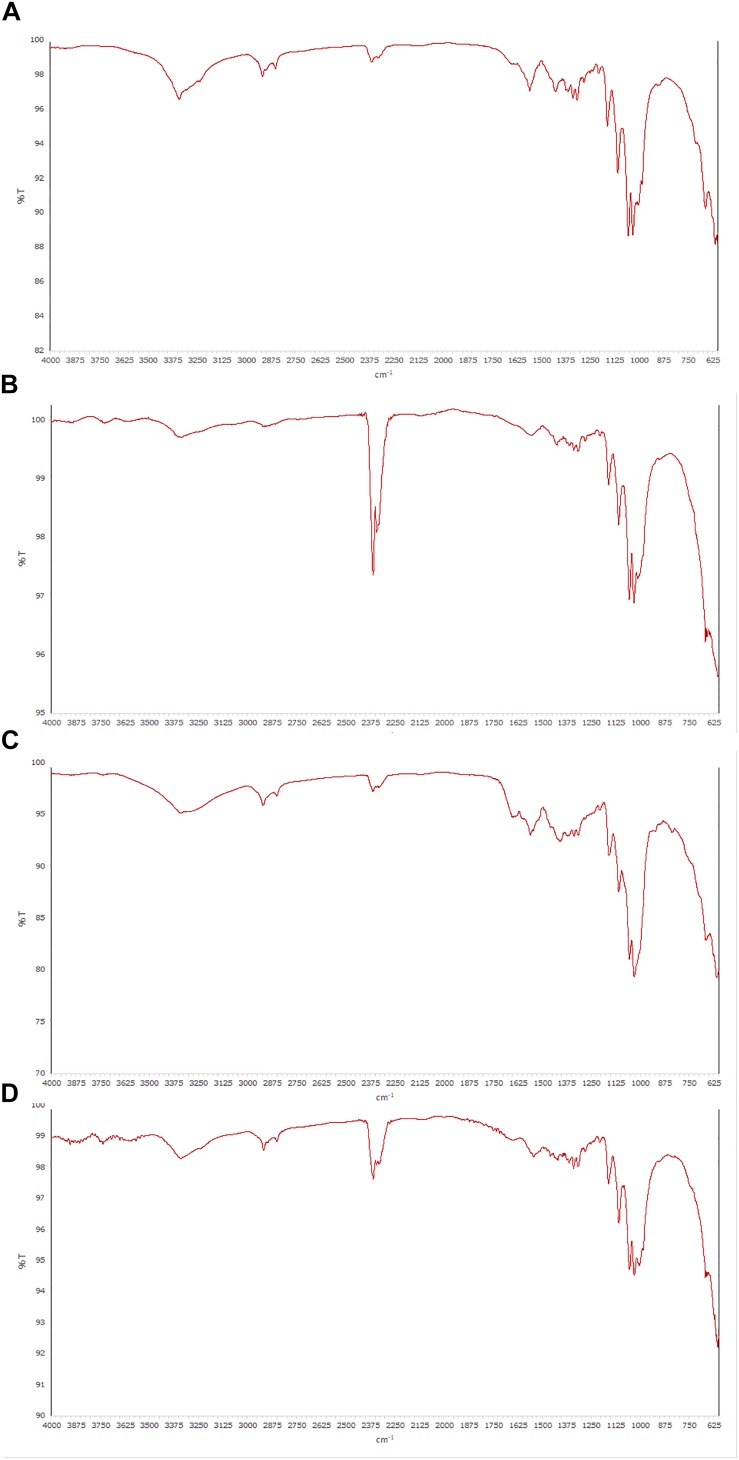
ATR-FT-IR spectra of cellulose samples produced from different strains. **(A,B)** GVP and GDP isolates of *K. rhaeticus* LMG 22126^T^ strain, respectively; **(C)** GSP isolate of *K. swingsii* LMG 22125^T^ strain; **(D)** *K. xylinus* LMG 1518 strain.

In accordance with previous works (Neera et al., 2015; Gullo et al., 2017; Thorat and Dastager, 2018; Chen et al., 2019), all spectra of the BC samples obtained by ATR-FT-IR spectroscopy, exhibited very similar profiles with “fingerprint” regions consistent with cellulose structure. The peak at 3350 cm^–1^ and the shouldering around 3400 cm^–1^ were due to the OH-stretching vibration, while the broad band at 2900 cm^–1^ could be assigned to the C-H stretching vibration (Lu and Jiang, 2014), showing the presence of amorphous cellulose. The signal at 1430 cm^–1^ that indicate a symmetric CH_2_ bending vibration, the occurrence of multiple absorption signals from 1600 to 950 cm^–1^ (1375 cm^–1^ C-H bending, 1335 cm^–1^ O-H in plane bending, 1315 cm^–1^ CH_2_ wagging, 1275 cm^–1^ C-H bending, 1225 cm^–1^ OH in plane, 1160 cm^–1^ C-O-C asymmetric stretching at the β-glycosidic linkage, 1060 cm^–1^ and 1030 cm^–1^ C-O stretching), as well as the absence of strong absorption band at 900 cm^–1^ (“amorphous” absorption band), confirmed a good crystalline organization for all specimens examined, as previously revealed by the results with NMR analysis. The ATR-FT-IR spectra of some cellulose samples are shown in Figure 4.

### Cellulose Yield Assessment

In our experimental conditions, the isolate GVP of *K. rhaeticus* LMG 22126^T^ was the highest producer of BC, while *K. hansenii* LMG 1527^T^ gave rise to a type of polymer with a promising rehydration capacity, potentially useful for food applications. As a result, these two strains were chosen to try to increase the yield of cellulose. The trials were performed as previously described. In Table 2, the cellulose amounts generated by the two strains after 5 days at 28°C in static conditions for each incubation step are reported. As expected, when the pH value of the medium went down, the synthesis of the polymer decreased. At the end of incubation, the sum of BC after 15 days was 9.7 g/L for isolate GVP of *K. rhaeticus* LMG 22126^T^ with a calculated carbon conversion rate of 22%, whereas that of *K. hansenii* LMG 1527^T^ was 3.6 g/L corresponding to 8%. The WHC values, measured at every step, were similar to those previously found with mean values of 22.5 and 26.9 (*g*_water_/*g*_cellulose_) for isolate GVP of *K. rhaeticus* LMG 22126^T^ and *K. hansenii* LMG 1527^T^, respectively. However, the rehydration capacity values were reduced by about one half compared to those formerly observed, with mean values of 2.5 and 4.0 (*g*_water_/*g*_cellulose_) for isolate GVP of *K. rhaeticus* LMG 22126^T^ and *K. hansenii* LMG 1527^T^, respectively.

**TABLE 2:** Amounts and characteristics of BC samples obtained by two selected isolates grown in static conditions at 28°C for 15 days, taking out the floating films every 5 days.

**Strain**	**Isolate**		**After 5 days**	**After 10 days**	**After 15 days**
*K. rhaeticus* LMG 22126^T^	GVP	Cellulose production (g/L)	4.7 ± 1.3	3.9 ± 2.5	1.1 ± 0.8
		pH of medium	4.0 ± 0.2	3.6 ± 0.3	3.4 ± 0.3
		Water holding capacity (*g*_water_/*g*_cellulose_)	22.1 ± 7.4	24.5 ± 5.5	20.9 ± 7.9
		Rehydration capacity (*g*_water_/*g*_cellulose_)	2.4 ± 1.4	2.8 ± 1.3	2.3 ± 0.9
*K. hansenii* LMG 1527^T^		Cellulose production (g/L)	2.4 ± 0.7	0.9 ± 0.3	0.3 ± 0.2
		pH of medium	4.2 ± 0.1	3.2 ± 0.2	3.0 ± 0.2
		Water holding capacity (*g*_water_/*g*_cellulose_)	23.8 ± 4.8	27.6 ± 4.6	29.2 ± 7.0
		Rehydration capacity (*g*_water_/*g*_cellulose_)	4.6 ± 0.6	4.4 ± 1.8	3.1 ± 1.3

*Values are mean of three replicates ± standard deviation.*

### SEM Observations and X-Ray Diffractometric Analysis

**FIGURE 5:**
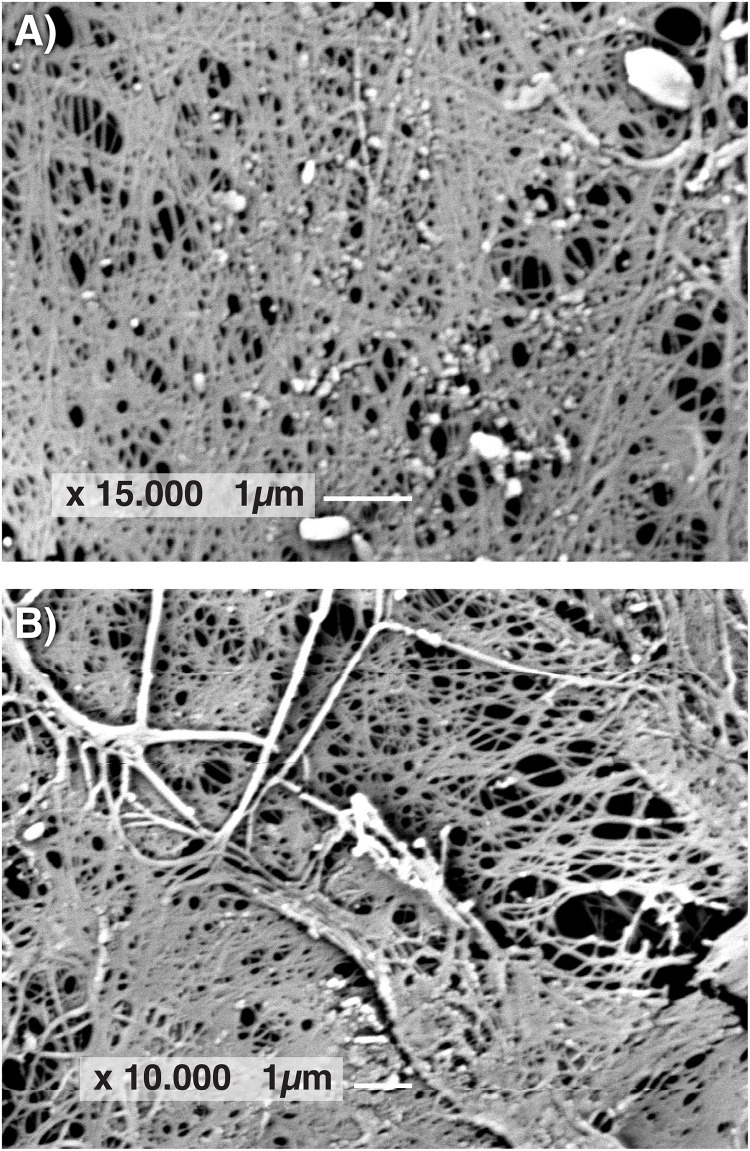
Scanning electron microscope images of BC samples from GVP isolate of *K. rhaeticus* LMG 22126^T^ before **(A)** and after purification **(B)**.

The morphology of BC films produced by GVP isolate of *K. rhaeticus* LMG 22126^T^ and *K. hansenii* LMG 1527^T^ was observed by SEM. In Figure 5, SEM images of cellulose samples from isolate GVP of *K. rhaeticus* LMG 22126^T^ before (A) and after purification (B) are shown. Micrographs revealed a three-dimensional network structure consisting of microfibrils with apparent diameters of 40–80 nm. Rod-shape bacterial cells of approximately 0.5 μm width and 1.0 μm long with attached cellulose ribbons are also visible (A).

In Supporting Information (Supplementary Figure S3), the XRD patterns of BC samples produced by isolate GVP of *K. rhaeticus* LMG 22126^T^ and *K. hansenii* LMG 1527^T^ are shown. The recorded diffractograms were similar to those of the purified native cellulose and in agreement with previous results on BC made by other species of the *Komagataeibacter* genus (Castro et al., 2011; Gullo et al., 2017; Molina-Ramírez et al., 2017). The three resolved peaks were assigned to (1 0 0), (0 1 0), and (1 1 0) crystallographic planes, corresponding to diffraction angles of 14.4°, 16.7°, and 22.6°, respectively, and indexed according to the cellulose Iα indexation described by Sugiyama et al. (1991). Specifically, XRD analysis supports the presence of the Iα form as major component, as previously supported by NMR analysis.

### Dough Leavening and Baking Trials for Gluten-Free Formulation With BC Addition

The effect of the addition of BC made by isolate GVP of *K. rhaeticus* LMG 22126^T^ was investigated in a gluten-free formulation by evaluating the dough leavening performances before and after baking. In the test samples, the cellulose was added during kneading as a hydrated gel, previously minced, by calculating the amount of dry BC which represented 1.25% w/w of the final dough, and taking into consideration the amount of water it contained to subtract from the total water needed for the recipe. The results were compared to ones obtained with other samples made with the same recipe without the BC addition (negative control) or done using a gluten-free flour mix with, approximately, the same quantity of cellulose (1.11% w/w, as HPMC, on the final dough) (positive control). For the three formulations, a dough consistency of 250 BU with a water absorption of 80% ± 1 was achieved. In Table 3, data regarding technological, chemical and microbiological characteristics of the loaves before and after baking are shown. Increases of 20% in dough height and of 26% gas retention coefficient were observed in samples with added BC in comparison with the negative control, proving that the polymer generated a structure which was able to sustain the dough development during proofing. Indeed, values of height in dough and bread samples made with the retailed formulation containing HPMC were comparable, confirming the role of cellulose in increasing and stabilizing the volume of the loaves. Surprisingly, the time of dough porosity appearance was significantly longer (*p* = 0.01), more than double, in samples prepared with BC compared to the samples without cellulose or those prepared with the gluten-free flour mix. Likewise, a strong change was detected in released CO_2_ volumes for the test samples in comparison with the negative or positive controls, although the differences were at less stringent significance level (*p* = 0.05). Furthermore, after 3 h of incubation, the pH values and the yeast counts in dough samples with BC addition were lower than those found in samples without BC or in samples containing HPMC (Table 3). As expected, the Acetic Acid Bacteria counts and both acetic and gluconic acid concentrations were significantly higher in dough samples with added BC compared to the negative and positive controls. Finally, loaves with added BC were characterized by lower crumb hardness, either at initial times and after 24 h, if likened to the other samples of bread. Also the crumb porosity was different showing that BC can improve the quality of final products.

**TABLE 3:** Leavening properties of the doughs, microbial counts and loaves characteristics in samples added or not with bacterial cellulose generated by GVP isolate of *K. rhaeticus* LMG 22126^T^ and in samples prepared with a gluten-free flour mix purchased at retail.

	**Samples with**		**Samples with**
	**added BC**	**Control**	**added HPMC**
Dough maximum height (mm)	23.8 ± 2.1	19.9 ± 3.9	23.9 ± 1.8
Dough height at the end of the test (mm)	22.3 ± 3.3	18.4 ± 4.1	23.9 ± 1.8
Time of dough porosity appearance (min)	59^a^ ± 11	29^b^ ± 8	27^b^ ± 3
CO_2_ total (mL)	2228 ± 273	2837 ± 171	2506 ± 289
CO_2_ rel (mL)	786 ± 261	1371 ± 175	1233 ± 35
CO_2_ ret (mL)	1442 ± 21	1466 ± 38	1453 ± 17
Gas retention coefficient (%)	65.4 ± 7.6	51.8 ± 3.4	54.1 ± 1.9
Final pH	4.36^a^ ± 0.08	4.67^b^ ± 0.06	4.69^b^ ± 0.06
Yeast counts (log CFU/g)	7.96^a^ ± 0.03	8.20^b^ ± 0.05	8.34^c^ ± 0.04
Acetic Acid Bacteria counts (log CFU/g)	6.17 ± 0.28	<3.00	<3.00
Acetic acid (g/L)	2.96^a^ ± 0.29	2.05^b^ ± 0.04	1.94^b^ ± 0.13
Gluconic acid (g/L)	5.21^a^ ± 1.08	0.04^b^ ± 0.01	0.03^b^ ± 0.01
Height of bread (mm)	41.2 ± 1.4	37.8 ± 1.6	36.3 ± 1.5
Firmness of crumb at initial time (N)	12.7^a^ ± 2.9	22.3^b^ ± 1.2	29.0^b^ ± 4.1
Firmness of crumb after 24 h (N)	39.8 ± 9.9	90.1 ± 41.1	58.9 ± 14.4

*Values are mean of three replicates ± standard deviation. Values with different superscripts of lowercase letter in the same row for each group of samples are significantly different (*p* < 0.01).*

## Discussion

The spontaneous formation of cellulose non-producing mutants were firstly reported for *A. xylinum* E_25_ strain by Krystynowicz et al. (2002) who mentioned that growth conditions caused a change in colony morphology. In 2005, Jung et al. (2005) studied the conversion of Cel+ cells to Cel− cells in *Gluconacetobacter hansenii* PJK strain by finding a relationship with the impeller speed in the bioreactor. Later, Fang and Catchmark (2015) monitored the induction of Cel− mutants in *G. hansenii* 53582, *Gluconacetobacter xylinus* 53524 and *G. xylinus* 700178 strains after transfer into shake-flask grown cultures. In this work, we observed the same phenomenon in *K. rhaeticus* and *K. swingsii* species, for the first time, confirming that BC productivity is linked to an environmental adaptation rather than a genotypic shuffling, since different clones derived from same strain showed indistinguishable RFLP DNA profiles. Even if the matter has not been definitively clarified, our results reveal that the isolated clones stably maintained their phenotypic characteristics if cultivated in static conditions and in a growth medium containing peptone.

Despite the low productivity of the process, BC has been successfully used in pharmaceutics, medicine, tissue engineering and regeneration, novel bio-materials, cosmetics and technological field for the development of high value-added products (Ullah et al., 2016; Bacakova et al., 2019; Hussain et al., 2019). This is not the case with food chains where the low profitability of the goods does not allow the use of BC as an ingredient due to its high price (Vandamme et al., 1998; Hussain et al., 2019). Moreover, while cellulose of bacterial origin is considered Generally Recognized As Safe (GRAS) by the U.S. Food and Drug Administration, the EFSA scientific panel has not yet decided on this (European Food Safety Authority [EFSA], 2018), so far making problematic the spread of this material for the food industry.

The choice of the carbon and nitrogen sources in the growth medium, the performance of a specific strain in terms of yield, the bio-reaction protocol and the design and setting-up of the plant have a strong impact on BC production costs (Chen et al., 2018). As already observed by several authors (Mikkelsen et al., 2009; Valera et al., 2015; Molina-Ramírez et al., 2017; Semjonovs et al., 2017; Volova et al., 2018; Chen et al., 2019), our outcome confirmed that glucose is the most useful substrate for increasing cellulose production, since it can work not only as an energy source but also as a direct cellulose precursor. Nevertheless, the pH decrease resulting from its conversion into gluconic acid dampened the BC yield, so that the higher buffer effect of peptone compared to ammonium sulfate or urea was decisive for enhancing the polymer production. However, in a recent paper Wang et al. (2018) summarized the results of many works on cellulose yield, concluding that the effect of the strain was the dominant one rather than the carbon source used. This conclusion has been corroborated by our findings where even isolates from sub-cultures of the same strain showed different productivity and behavior, though the structural characteristics of the cellulose synthesized were quite similar. Therefore, the selection of mutants may be more important than the choice of the cultural medium formulation in order to design and set-up an efficient BC production under the wanted conditions. Moreover, this work demonstrates that *K. rhaeticus* in addition to the well-known *K. xylinus* can be considered a very performing species for the synthesis of cellulose, as already emphasized by Semjonovs et al. (2017) and Thorat and Dastager (2018).

The solid state NMR spectra provided important evidences concerning the basic structure of cellulose samples, related to the conformational differences between the polymorphs and the crystalline and/or amorphous nature of the polymer (Foston, 2014). Based on ^13^C chemical shifts, all BC samples consisted of two crystalline allomorphs Iα and Iβ in different ratios. The experiments afforded resolved profiles with well-defined peaks for the crystalline forms and very broad signals for the amorphous ones; the assignments were in agreement with the literature data (Kamide, 2005). Irrespective of the different strains, the cellulose produced exhibited a high level of crystallinity, with a dominance for Iα fraction. It is worth noting, in cellulose samples deriving from two isolates of *K. swingsii* and *K. nataicola*, that signals of esterified groups were detected, which could be potential targets for a polymer functionalization. FT-IR spectroscopy has established the cellulosic structure of the polymer without showing any difference from spectra reported in the literature (Neera et al., 2015; Gullo et al., 2017; Thorat and Dastager, 2018; Chen et al., 2019).

As regards the BC productivity, results obtained from isolate GVP of *K. rhaeticus* LMG 22126^T^ have been encouraging and in agreement with the highest values recently described and reviewed by Hussain et al. (2019) for other strains of acetic acid bacteria. Furthermore, the carbon conversion rate for the above-mentioned isolate was very similar to that described by Semjonovs et al. (2017) and more than double those reported by Chen et al. (2017) for *K. hansenii* and *K. xylinus* strains. On the contrary, some authors argue that higher conversion levels can be obtained with glycerol as the carbon source (Keshk and Sameshima, 2005; Zhong et al., 2013; Tabaii and Emtiazi, 2016; Zhang et al., 2017; Thorat and Dastager, 2018), since it is consumed into the Krebs cycle without the accumulation of gluconic acid in the growth medium.

Great water-absorbing and -holding aptitudes are desirable properties of BC for its potential uses in food applications (Ullah et al., 2016; Gomes et al., 2018). On average, WHC values of cellulose samples formed by the strains investigated in this work were higher than those reported in other papers (Chau et al., 2008; Chen et al., 2018), though data relating to the WHC of *Komagataeibacter* genus are not numerous and those relating to the rehydration capacity are even more scarce (Zhang et al., 2018). We examined BC samples for these characteristics by considering them valuable for a technological addition to foods that require moisture retention and thickening properties.

Looking at our findings, we thought it was more effective to use minced hydrated gels containing BC, as a structuring agent in gluten-free dough formulations, rather than dry BC since the polymer re-hydration capacity has been demonstrated to be lower than the original WHC. In dough leavening and baking trials, the rheological characteristics of the samples prepared with the addition of wet BC were better than those of the control samples. The presence of BC and *Komagataeibacter* cells affected the growth of the yeast population, causing a slowdown of fermentation, probably due to the light inhibiting activity of acetic acid on *Saccharomyces cerevisiae*. However, this has turned out in an advantage in terms of less gas release and more CO_2_ retention during the dough leavening and better development of the loaves after baking. The use of minced hydrated gels have the advantage to retain the acidity of the dough, mainly of gluconic and acetic acids, with a positive sensory impact and natural antagonistic effects against the development of molds during the shelf life of the product.

The interest in Acetic Acid Bacteria in food systems is growing (Sengun and Karabiyikli, 2011) because they prove to be efficient producers of pure cellulose, the economic use of which depends on the reduction of operative costs (Ul-Islam et al., 2017). Molecular mechanisms underlying the expression of genes for cellulose synthesis in *Komagataeibacter* spp. strains have to be well elucidated yet, although certain studies have been focussed on them (Augimeri and Strap, 2015; Wang et al., 2018). In order to optimize a BC large-scale production Gullo et al. (2019) recently proposed a strategy that integrate information deriving from technological and genomic data. As reviewed by Hussain et al. (2019), the use of low-cost agro and food industrial wastes as raw materials for this purpose would make sense and help to develop a new market in a circular economy perspective.

## Data Availability

The raw data supporting the conclusions of this manuscript will be made available by the authors, without undue reservation, to any qualified researcher.

## Author Contributions

IV contributed to the design of the work, to manage the lab work, to collect and elaborate the data, and to draft the manuscript. VF contributed to perform the microbiological and chemical analyses, and to draft the work. FD contributed to perform the microbiological, chemical and technological analyses, and to draft the work. SR contributed to perform the chemical and spectroscopy analyses, the SEM observations, and to draft the work. IA contributed to perform the molecular analysis. CM contributed to make dough leavening and baking trials, and to perform the technological analyses. MB contributed to the design of the work, to manage the lab work, to collect and elaborate data, to draft and review the manuscript. RF contributed to the design and organization of the work, to data collection and analysis, to draft and review the manuscript, and to ensure that all questions related to the accuracy or integrity of any part of the work were appropriately investigated and resolved.

## Conflict of Interest Statement

VF and IA were employed by company LaVitaWiz, Wiz Chemicals. CM was employed by company Farine Varvello & Co. The remaining authors declare that the research was conducted in the absence of any commercial or financial relationships that could be construed as a potential conflict of interest.

## Supplementary Material

FIGURE S1 DNA restriction patterns of LMG 22126^T^ *K. rhaeticus* and LMG 22125^T^ *K. swingsii* strains generated by *Xba*I in PFGE analysis. Line 1 isolate GDG, line 2 isolate GDP, line 3 isolate GVP, line 4 isolate GSG, and line 5 isolate GSP.

FIGURE S2 ^13^C CP/MAS NMR spectrum **(A)** and ^1^H CP/MAS NMR spectrum **(B)** of bacterial cellulose synthesized by the isolate GSP of *K. swingsii* LMG 22125^T^.

FIGURE S3 X-ray diffraction spectra of BC samples produced by GVP isolate of *K. rhaeticus* LMG 22126^T^ (black line) and *K. hansenii* LMG 1527^T^ (blue line).

TABLE S1 Mean values ± standard deviation of optical sensity, final pH in growth medium, and BC production for nine *Komagataibacter* spp. isolates after cultivation with different carbon and nitrogen sources at 28°C for 5 days in static conditions.

## References

[B1] Augimeri R. V. Strap J. L. (2015). The phytohormone ethylene enhances cellulose production, regulates CRP/FNRKx transcription and causes differential gene expression within the bacterial cellulose synthesis operon of *Komagataeibacter* (*Gluconacetobacter*) *xylinus* ATCC 53582. *Front. Microbiol.* 6:1459. 10.3389/fmicb.2015.01459 26733991 PMC4686702

[B2] Augimeri R. V. Varley A. J. Strap J. L. (2015). Establishing a role for bacterial cellulose in environmental interactions: lessons learned from diverse biofilm-producing *Proteobacteria*. *Front. Microbiol.* 6:1282. 10.3389/fmicb.2015.01282 26635751 PMC4646962

[B3] Azeredo H. M. C. Barud H. Farinas C. S. Vasconcellos V. M. Claro A. M. (2019). Bacterial cellulose as a raw material for food and food packaging applications. *Front. Sustain. Food Syst.* 3:7 10.3389/fsufs.2019.00007

[B4] Bacakova L. Pajorova J. Bacakova M. Skogberg A. Kallio P. Kolarova K. (2019). Versatile application of nanocellulose: from industry to skin tissue engineering and wound healing. *Nanomaterials* 9:E164. 10.3390/nano9020164 30699947 PMC6410160

[B5] Bourdichon F. Casaregola S. Farrokh C. Frisvad J. C. Gerds M. L. Hammes W. P. (2012). Food fermentations: microorganisms with technological beneficial use. *Int. J. Food Microbiol.* 154 87–97. 10.1016/j.ijfoodmicro.2011.12.030 22257932

[B6] Campano C. Balea A. Blanco A. Negro C. (2016). Enhancement of the fermentation process and properties of bacterial cellulose: a review. *Cellulose* 23 57–91. 10.1007/s10570-015-0802-0

[B7] Castro C. Zuluaga R. Putaux J. L. Caro G. Mondragon I. Gaňán P. (2011). Structural characterization of bacterial cellulose produced by *Gluconacetobacter swingsii* sp. from Colombian agroindustrial wastes. *Carbohydr. Polym.* 84 96–102. 10.1016/j.carbpol.2010.10.072

[B8] Chau C. F. Yang P. Yu C. M. Yen G. C. (2008). Investigation on the lipid- and cholesterol lowering abilities of biocellulose. *J. Agric. Food Chem.* 56 2291–2295. 10.1021/jf7035802 18318496

[B9] Chen G. Wu G. Chen L. Wang W. Hong F. F. Jönsson L.-J. (2019). Comparison of productivity and quality of bacterial nanocellulose synthesized using culture media based on seven sugars from biomass. *Microb. Biotechnol.* 12 677–687. 10.1111/1751-7915.13401 30912251 PMC6559334

[B10] Chen S.-Q. Lopez-Sanchez P. Wang D. Mikkelsen D. Gidley M. J. (2018). Mechanical properties of bacterial cellulose synthesised by diverse strains of the genus *Komagataeibacter*. *Food Hydrocoll.* 81 87–95. 10.1016/j.foodhyd.2018.02.031

[B11] Chen S.-Q. Mikkelsen D. Lopez-Sanchez P. Wang D. Martinez-Sanz M. Gilbert E. P. (2017). Characterisation of bacterial cellulose from diverse *Komagataeibacter* strains and their application to construct plant cell wall analogues. *Cellulose* 24 1211–1226. 10.1007/s10570-017-1203

[B12] Commission Regulation (2012). (EU) No 231/2012 of 9 March 2012 laying down specifications for food additives listed in Annexes II, and III to regulation (EC)No 1333/2008 of the European parliament and of the council. *Off. J. Eur. Union L* 83 1–295.

[B13] De Roos J. De Vuyst L. (2018). Acetic acid bacteria in fermented foods and beverages. *Curr. Opin. Biotechnol.* 49 115–119. 10.1016/j.copbio.2017.08.007 28863341

[B14] Dellaglio F. Cleenwerck I. Felis G. E. Engelbeen K. Janssens D. Marzotto M. (2005). Description of *Gluconacetobacter swingsii* sp. nov. and *Gluconacetobacter rhaeticus* sp. nov., isolated from Italian apple fruit. *Int. J. Syst. Evol. Microbiol.* 55 2365–2370. 10.1099/ijs.0.63301-0 16280498

[B15] Dourado F. Gama M. Rodrigues A. C. (2017). A review on the toxicology and dietetic role of bacterial cellulose. *Toxicol. Rep.* 4 543–553. 10.1016/j.toxrep.2017.09.005 29090119 PMC5655389

[B16] European Food Safety Authority [EFSA] (2018). *Draft Minutes of the 85th Plenary Meeting. Request for a Scientific Opinion on Bacterial Cellulose Aqueous Suspension as a Novel Food (NF 2018/0307).* Parma: European Food Safety Authority.

[B17] European Food Safety Authority (2019). Update of the list of QPS-recommended biological agents intentionally added to food or feed as notified to EFSA 9: suitability of taxonomic units notified to EFSA until September 2018. *EFSA J.* 17:e05555. 10.2903/j.efsa.2019.5555 32626100 PMC7328880

[B18] Fang L. Catchmark J. M. (2015). Characterization of cellulose and other exopolysaccharides produced from *Gluconacetobacter* strains. *Carbohydr. Polym.* 115 663–669. 10.1016/j.carbpol.2014.09.028 25439946

[B19] Federal Institute for Occupational Safety and Health (2010). *Classification of Prokaryotes (Bacteria and Archaea) into Risk Groups TRBA 466.* Germani: Federal Institute for Occupational Safety, and Health.

[B20] Foston M. (2014). Advances in solid-state NMR of cellulose. *Curr. Opin. Biotechnol.* 27 176–184. 10.1016/j.copbio.2014.02.002 24590189

[B21] Gallegos A. M. A. Carrera S. H. Parra R. Keshavarz T. Iqbal H. M. N. (2016). Bacterial cellulose: a sustainable source to develop value-added products-a review. *BioResearch* 11 5641–5655.

[B22] Gomes R. J. Borges M. F. Rosa M. F. Castro-Gómez R. J. H. Spinosa W. A. (2018). Acetic Acid Bacteria in the food industry: systematics, characteristics and applications. *Food Technol. Biotechnol.* 56 139–151. 10.17113/ftb.56.02.18.5593 30228790 PMC6117990

[B23] Gulitz A. Stadie J. Wenning M. Ehrmann M. A. Vogel R. F. (2011). The microbial diversity of water kefir. *Int. J. Food Microbiol.* 151 284–288. 10.1016/j.ijfoodmicro.2011.09.016 22000549

[B24] Gullo M. Giudici P. (2008). Acetic acid bacteria in traditional balsamic vinegar: phenotypic traits relevant for starter cultures selection. *Int. J. Food Microbiol.* 125 46–53. 10.1016/j.ijfoodmicro 18177968

[B25] Gullo M. La China S. Falcone P. M. Giudici P. (2018). Biotechnological production of cellulose by acetic acid bacteria: current state and perspectives. *Appl. Microbiol. Biotechnol.* 102 6885–6898. 10.1007/s00253-018-9164-5 29926141

[B26] Gullo M. La China S. Petroni G. Di Gregorio S. Giudici P. (2019). Exploring K2G30 genome: a high bacterial cellulose producing strain in glucose and mannitol based media. *Front. Microbiol.* 10:58. 10.3389/fmicb.2019.00058 30761107 PMC6363697

[B27] Gullo M. Sola A. Zanichelli G. Montorsi M. Messori M. Giudici P. (2017). Increased production of bacterial cellulose as starting point for scaled-up applications. *Appl. Microbiol. Biotechnol.* 101 8115–8127. 10.1007/s00253-017-8539-3 28965208

[B28] Hussain Z. Sajjad W. Wahid F. (2019). Production of bacterial cellulose from industrial wastes: a review. *Cellulose* 26 2895–2911. 10.1007/s10570-019-02307-1

[B29] Idström A. Schants S. Sundberg J. Chmlka B. F. Gatenholm P. Nordstierna L. (2016). 13C NMR assignments of regenerated cellulose from solid-state 2D NMR spectroscopy. *Carbohydr. Polym.* 151 480–487. 10.1016/j.carbpol.2016.05.107 27474592

[B30] Jagannath A. Raju P. S. Bawa A. S. (2010). Comparative evaluation of bacterial cellulose (nata) as a cryoprotectant and carrier support during the freeze drying process of probiotic lactic acid bacteria. *Food Sci. Technol.* 43 1197–1203. 10.1016/j.lwt.2010.03.009

[B31] Jung J. Y. Park J. K. Chang H. N. (2005). Bacterial cellulose production by *Gluconacetobacter hansenii* in an agitated culture without living non-cellulose producing cells. *Enz. Microb. Technol.* 37 347–354. 10.1016/j.enzmictec.2005.02.019

[B32] Kamide K. (2005). *Cellulose and Cellulose Derivatives Molecular Characterization and its Applications.*, 1st Edn Amsterdam: Elsevier Science.

[B33] Keshk S. M. A. S. Sameshima K. (2005). Evaluation of different carbon sources for bacterial cellulose production. *Afr. J. Biotechnol.* 4 478–482.

[B34] Krystynowicz A. Czaja W. Wiktorowska-Jezierska A. Gonçalves-Miskiewicz M. Turkiewicz M. Bielecki S. (2002). Factors affecting the yield and properties of bacterial cellulose. *J. Ind. Microbiol. Biotechnol.* 29 189–195. 10.1038/sj/jim/7000303 12355318

[B35] Lu H. Jiang X. (2014). Structure and properties of bacterial cellulose produced using a trickling bed reactor. *Appl. Biochem. Biotechnol.* 172 3844–3861. 10.1007/s12010-014-0795-4 24682876

[B36] Mikkelsen D. Flanagan B. M. Dykes G. A. Gidley M. J. (2009). Influence of different carbon sources on bacterial cellulose production by *Gluconacetobacter xylinus* strain ATCC 53524. *J. Appl. Microbiol.* 107 576–583. 10.1111/j.1365-2672.2009.04226.x 19302295

[B37] Molina-Ramírez C. Castro M. Osorio M. Torres-Taborda M. Gómez B. Zuluaga R. (2017). Effect of different carbon sources on bacterial nanocellulose production and structure using the low pH resistant strain *Komagataeibacter medellinensis*. *Materials* 10:639. 10.3390/ma10060639 28773001 PMC5554020

[B38] Moon R. J. Schueneman G. T. Simonsen J. (2016). Overview of cellulose nanomaterials, their capabilities and applications. *JOM* 68 2383–2394. 10.1007/s11837-016-2018-7

[B39] Neera, Ramana K. V. Batra H. V. (2015). Occurrence of cellulose-producing *Gluconacetobacter* spp. in fruit samples and kombucha tea, and production of the biopolymer. *Appl. Biochem. Biotechnol.* 176 1162–1173. 10.1007/s12010-015-1637 25926011

[B40] Park S. Baker J. O. Himmel M. E. Parilla P. A. Johnson D. K. (2010). Cellulose crystallinity index: measurement techniques and their impact on interpreting cellulase performance. *Biotechnol. Biofuels* 3 1–10. 10.1186/1754-6834-3-10 20497524 PMC2890632

[B41] Picozzi C. Bonacina G. Vigentini I. Foschino R. (2010). Genetic diversity in Italian *Lactobacillus sanfranciscensis* strains assessed by multilocus sequence typing and pulsed-field gel electrophoresis analyses. *Microbiology* 156 2035–2045. 10.1099/mic.0.037341-0 20360177

[B42] Rai S. Kaur A. Chopra C. S. (2018). Gluten-free products for celiac susceptible people. *Front. Nutr.* 5:116. 10.3389/fnut.2018.00116 30619866 PMC6304385

[B43] Rueda J. C. Komber H. Cedron J. C. Voit B. Shevtsova G. (2003). Synthesis of new hydrogels by copolymerization of poly(2-methyl-2-oxazoline) bis (macromonomers) and N-vinylpyrrolidone. *Macromol. Chem. Phys.* 204 947–953. 10.1002/macp.200390062

[B44] Semjonovs P. Ruklisha M. Paegle L. Saka M. Treimane R. Skute M. (2017). Cellulose synthesis by *Komagataeibacter rhaeticus* strain P 1463 isolated from Kombucha. *Appl. Microbiol. Biotechnol.* 101 1003–1012. 10.1007/s00253-016-7761-8 27678116

[B45] Sengun I. Y. Karabiyikli S. (2011). Importance of acetic acid bacteria in food industry. *Food Control* 22 647–656. 10.1016/j.foodcont.2010.11.008

[B46] Singhsa P. Narain R. Manuspiya H. (2018). Physical structure variations of bacterial cellulose produced by different *Komagataeibacter xylinus* strains and carbon sources in static and agitated conditions. *Cellulose* 25 1571–1581. 10.1007/s10570-018-1699-1

[B47] Sugiyama J. Persson J. Chanzy H. (1991). Combined infrared and electron diffraction study of the polymorphism of native celluloses. *Macromology* 24 2461–2466. 10.1021/ma00009a050

[B48] Sun M. Wang Y. Shia L. Klemeš J. J. (2018). Uncovering energy use, carbon emissions and environmental burdens of pulp and paper industry: a systematic review and meta-analysis. *Renew Sustain. En. Rev.* 92 823–833. 10.1016/j.rser.2018.04.036

[B49] Štornik A. Skok B. Trček J. (2016). Bacterial microbiota in apple cider vinegar. *Food Technol. Biotechnol.* 54 113–119. 10.17113/b.54.01.16.4082 27904401 PMC5105631

[B50] Tabaii M. J. Emtiazi G. (2016). Comparison of bacterial cellulose production among different strains and fermented media. *Appl. Food Biotechnol.* 3 35–41.

[B51] Thorat M. N. Dastager S. G. (2018). High yield production of cellulose by a *Komagataeibacter rhaeticus* PG2 strain isolated from pomegranate as a new host. *Royal Society Chem. Adv.* 8 29797–29805. 10.1039/c8ra05295f PMC9085265 35547325

[B52] Ul-Islam M. Ullah M. W. Khan S. Shah N. Park J. K. (2017). Strategies for cost-effective and enhanced production of bacterial cellulose. *Int. J. Biol. Macromol.* 102 1166–1173. 10.1016/j.ijbiomac.2017.04.110 28487196

[B53] Ullah H. Santos H. A. Khan T. (2016). Applications of bacterial cellulose in food, cosmetics and drug delivery. *Cellulose* 2 32291–32314. 10.1007/s10570-016-0986-y

[B54] Valera M. J. Torija M. J. Mas A. Mateo E. (2015). Cellulose production and cellulose synthase gene detection in acetic acid bacteria. *Appl. Microbiol. Biotechnol.* 99 1349–1361. 10.1007/s00253-014-6198-1 25381910

[B55] Vandamme E. J. De Baets S. Vanbaelen A. Joris K. De Wulf P. (1998). Improved production of bacterial cellulose and its application potential. *Polym. Degrad. Stab.* 59 93–99. 10.1016/s0141-3910(97)00185-7

[B56] Villarreal-Soto S. A. Beaufort S. Bouajila J. Souchard J.-P. Taillandier P. (2018). Understanding kombucha tea fermentation: a review. *J. Food Sci.* 83 580–588. 10.1111/1750-3841.14068 29508944

[B57] Volova T. G. Prudnikova S. V. Sukovatyi A. G. Shishatskaya E. I. (2018). Production and properties of bacterial cellulose by the strain *Komagataeibacter xylinus* B-12068. *Appl. Microbiol. Biotechnol.* 102 7417–7428. 10.1007/s00253-018-9198 29982923

[B58] Wang S.-S. Han Y.-H. Chen J.-L. Zhang D.-C. Shi X.-X. Ye Y.-X. (2018). Insights into bacterial cellulose biosynthesis from different carbon sources and the associated biochemical transformation pathways in *Komagataeibacter* sp. W1. *Polymers* 10:963. 10.3390/polym10090963 30960888 PMC6403882

[B59] Yamada Y. Yukphan P. Thi Lan Vu W. Muramatsu Y. Ochaikul D. Tanasupawat S. (2012). Description of *Komagataeibacter* gen. nov., with proposals of new combinations (Acetobacteraceae). *J. Gen. Appl. Microbiol.* 58 397–404. 23149685 10.2323/jgam.58.397

[B60] Zhang J. Yang Y. Deng J. Wang Y. Hu Q. Li C. (2017). Dynamic profile of the microbiota during coconut water pre-fermentation for nata de coco production. *Food Sci. Technol.* 81 87–93. 10.1016/j.lwt.2017.03.036

[B61] Zhang W. Wang X. Qi X. Ren L. Qiang T. (2018). Isolation and identification of a bacterial cellulose synthesizing strain from kombucha in different conditions: *Gluconacetobacter xylinus* ZHCJ618. *Food Sci. Biotechnol.* 27 705–713. 10.1007/s10068-018-0303-7 30263796 PMC6049678

[B62] Zhong C. Zhang G.-C. Liu M. Zheng X.-T. Han P.-P. Jia S.-R. (2013). Metabolic flux analysis of *Gluconacetobacter xylinus* for bacterial cellulose production. *Appl. Microbiol. Biotechnol.* 97 6189–6199. 10.1007/s00253-013-4908 23640364

