# Supplementary figures and images for: Set-Up of Bacterial Cellulose Production From the Genus Komagataeibacter and Its Use in a Gluten-Free Bakery Product as a Case Study
Source: Front Microbiol. 2019 Sep 6;10:1953. doi: 10.3389/fmicb.2019.01953 (PMC6743508; doi:10.3389/fmicb.2019.01953)

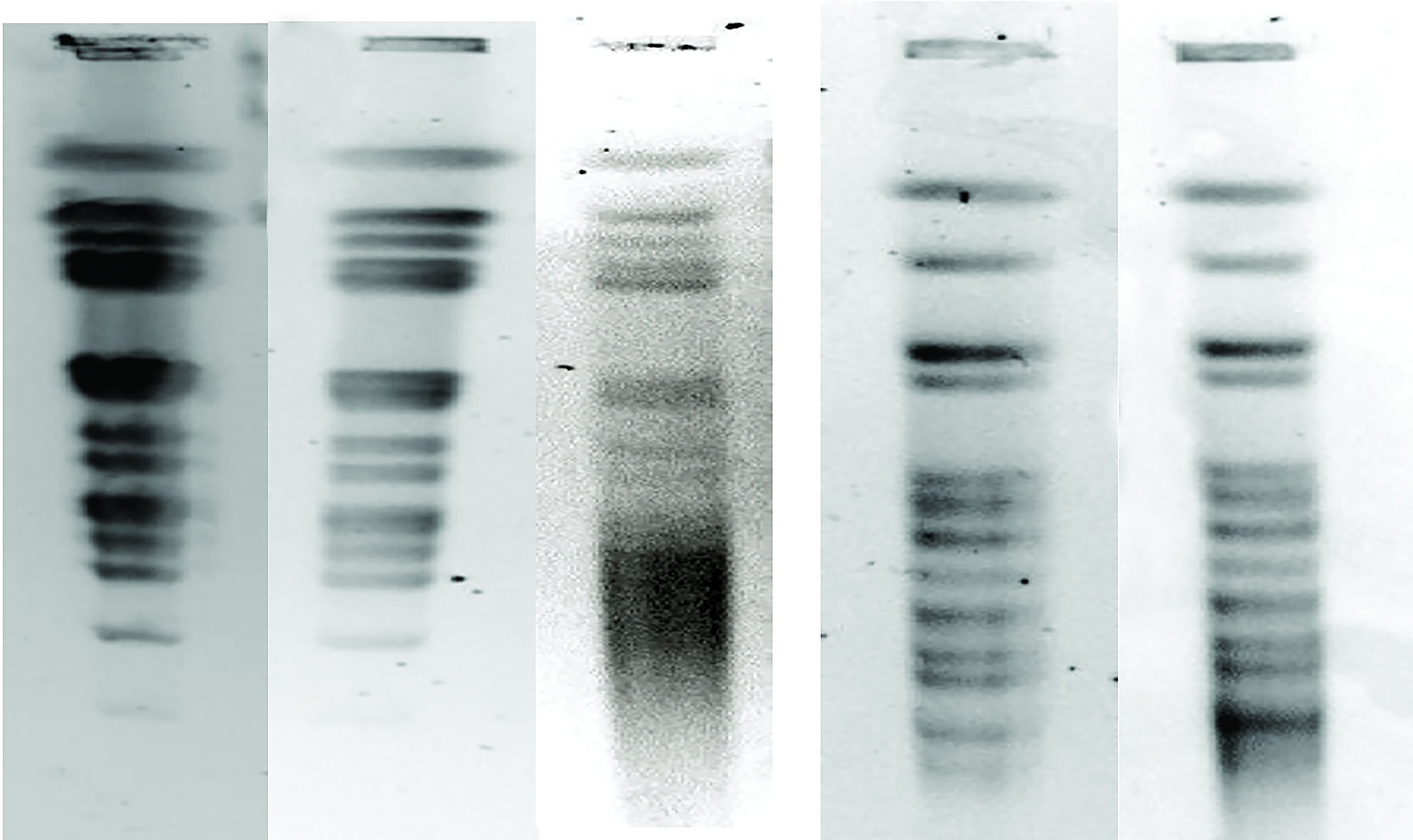

Supplement: FIGURE S1 — DNA restriction patterns of LMG 22126T K. rhaeticus and LMG 22125T K. swingsii strains generated by XbaI in PFGE analysis. Line 1 isolate GDG, line 2 isolate GDP, line 3 isolate GVP, line 4 isolate GSG, and line 5 isolate GSP. [file Image_1.TIF]

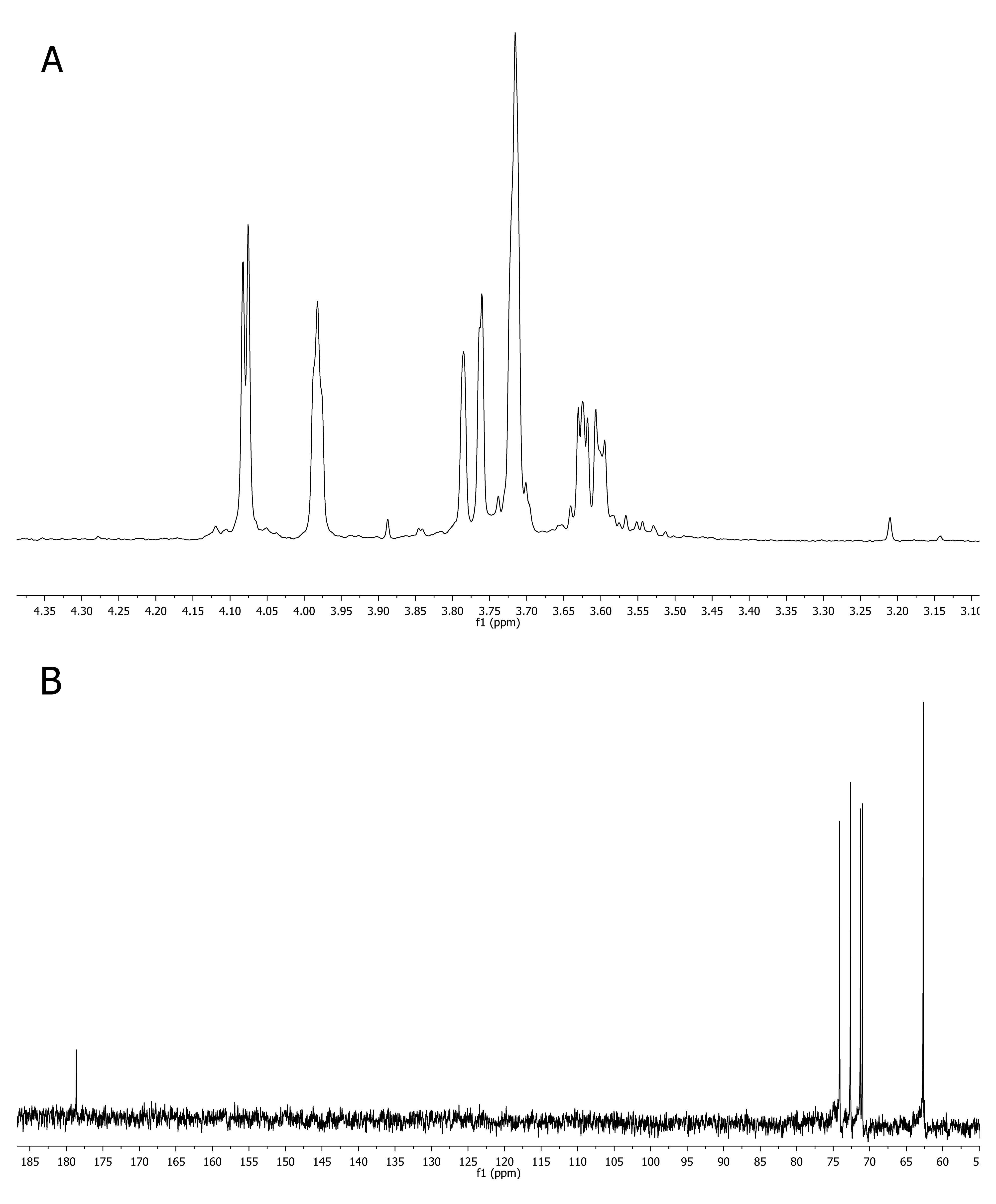

Supplement: FIGURE S2 — 13C CP/MAS NMR spectrum (A) and 1H CP/MAS NMR spectrum (B) of bacterial cellulose synthesized by the isolate GSP of K. swingsii LMG 22125T. [file Image_2.TIF]

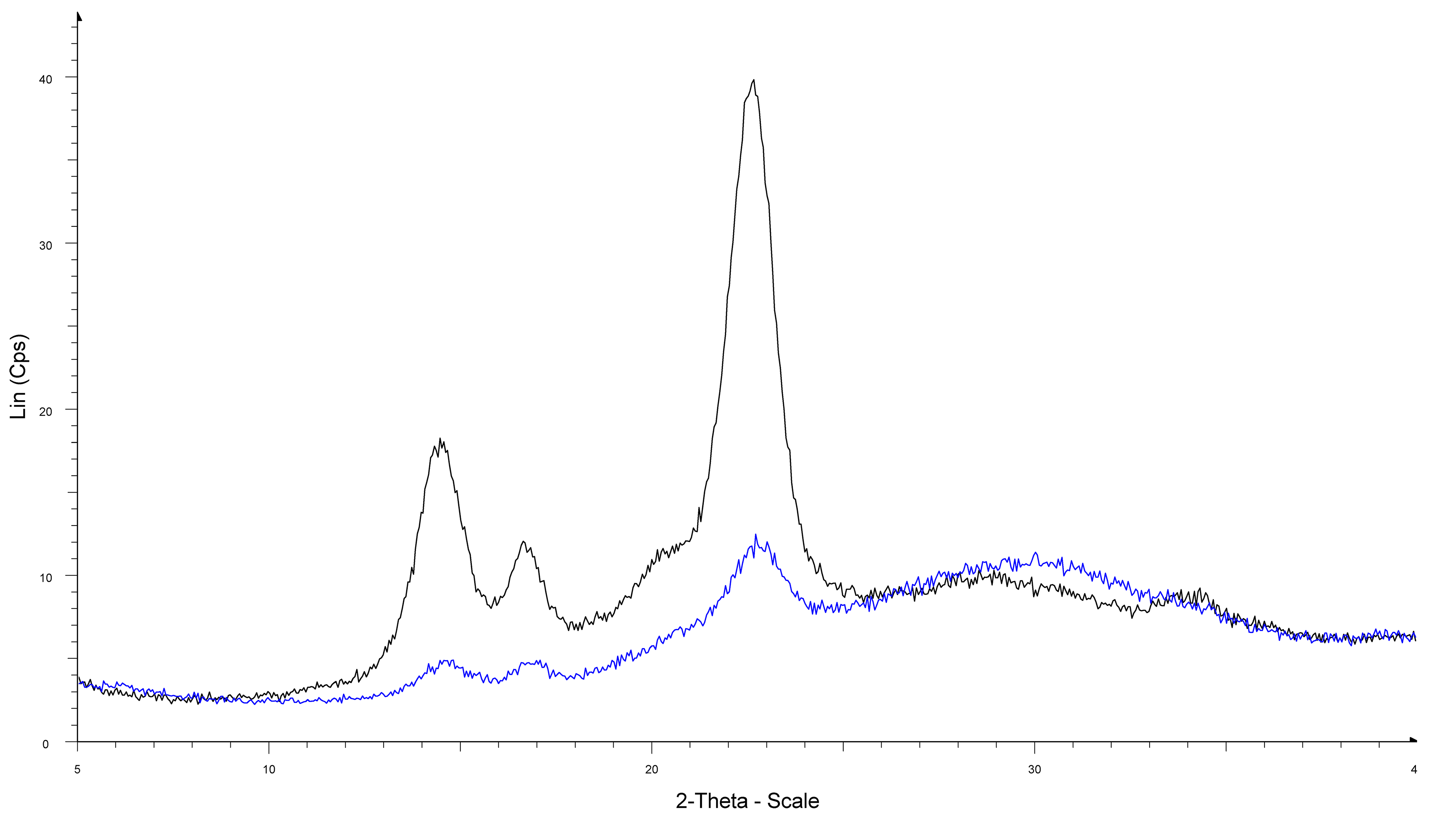

Supplement: FIGURE S3 — X-ray diffraction spectra of BC samples produced by GVP isolate of K. rhaeticus LMG 22126T (black line) and K. hansenii LMG 1527T (blue line). [file Image_3.TIF]
