# Supplementary material for: Set-Up of Bacterial Cellulose Production From the Genus Komagataeibacter and Its Use in a Gluten-Free Bakery Product as a Case Study
Source: Front Microbiol. 2019 Sep 6;10:1953. doi: 10.3389/fmicb.2019.01953 (PMC6743508; doi:10.3389/fmicb.2019.01953)
Supplement: TABLE S1 — Mean values ± standard deviation of optical sensity, final pH in growth medium, and BC production for nine Komagataibacter spp. isolates after cultivation with different carbon and nitrogen sources at 28°C for 5 days in static conditions. [file Table_1.docx]

**Table 1S**. Mean values ± standard deviation of Optical Density, final pH in growth medium and BC production for nine *Komagataibacter* spp. isolates after cultivation with different carbon and nitrogen sources at 28 °C for 5 days in static conditions.

| Species | Isolate | C source | N source | OD_600nm_ | pH | Cellulose (g/L) |
| --- | --- | --- | --- | --- | --- | --- |
| *K. hansenii* LMG 1527 |  | glucose | ammonium | 0.28 ± 0.1 | 2.74 ± 0.1 | < 0.50 |
|  |  |  | urea | 0.23 ± 0.1 | 3.23 ± 0.5 | 0.81 ± 0.4 |
|  |  |  | peptone | 0.07 ± 0.1 | 4.05 ± 0.1 | 1.84 ± 0.2 |
|  |  | fructose | ammonium | 0.33 ± 0.1 | 4.48 ± 0.8 | < 0.50 |
|  |  |  | urea | 0.17 ± 0.1 | 4.85 ± 0.9 | < 0.50 |
|  |  |  | peptone | 0.09 ± 0.1 | 6.09 ± 0.1 | 2.88 ± 0.5 |
|  |  | sucrose | ammonium | 0.27 ± 0.1 | 5.40 ± 1.6 | < 0.50 |
|  |  |  | urea | 0.13 ± 0.1 | 5.68 ± 1.7 | 1.25 ± 0.7 |
|  |  |  | peptone | 0.04 ± 0.0 | 7.18 ± 0.1 | 1.50 ± 0.2 |
|  |  | maltose | ammonium | 0.28 ± 0.1 | 4.57 ± 0.8 | < 0.50 |
|  |  |  | urea | 0.14 ± 0.1 | 5.35 ± 1.4 | 1.53 ± 1.2 |
|  |  |  | peptone | 0.10 ± 0.1 | 6.89 ± 0.1 | 1.59 ± 0.1 |
|  |  | glycerol | ammonium | 0.34 ± 0.1 | 4.88 ± 0.9 | < 0.50 |
|  |  |  | urea | 0.24 ± 0.1 | 6.02 ± 2.0 | 0.90 ± 0.8 |
|  |  |  | peptone | 0.14 ± 0.1 | 6.87 ± 0.1 | 2.56 ± 0.1 |
| *K. nataicola* LMG 1536 |  | glucose | ammonium | 0.28 ± 0.1 | 2.91 ± 0.2 | 0.93 ± 0.6 |
|  |  |  | urea | 0.16 ± 0.1 | 4.73 ± 0.1 | 0.80 ± 0.7 |
|  |  |  | peptone | 0.44 ± 0.1 | 3.86 ± 0.1 | 7.51 ± 0.3 |
|  |  | fructose | ammonium | 0.27 ± 0.1 | 3.37 ± 0.1 | 1.58 ± 1.1 |
|  |  |  | urea | 0.17 ± 0.1 | 5.41 ± 1.7 | 1.85 ± 1.8 |
|  |  |  | peptone | 0.21 ± 0.2 | 5.45 ± 0.1 | 3.88 ± 0.3 |
|  |  | sucrose | ammonium | 0.31 ± 0.2 | 3.42 ± 0.1 | 2.80 ± 2.7 |
|  |  |  | urea | 0.15 ± 0.1 | 5.54 ± 1.7 | 0.75 ± 0.1 |
|  |  |  | peptone | 0.09 ± 0.0 | 7.16 ± 0.1 | 6.99 ± 0.3 |
|  |  | maltose | ammonium | 0.29 ± 0.1 | 3.42 ± 0.1 | 1.51 ± 1.2 |
|  |  |  | urea | 0.25 ± 0.1 | 5.86 ± 2.2 | 2.57 ± 1.5 |
|  |  |  | peptone | 0.10 ± 0.0 | 7.18 ± 0.1 | 0.78 ± 0.3 |
|  |  | glycerol | ammonium | 0.14 ± 0.1 | 3.76 ± 0.1 | 0.51 ± 0.1 |
|  |  |  | urea | 0.26 ± 0.2 | 6.07 ± 2.1 | < 0.50 |
|  |  |  | peptone | 0.10 ± 0.0 | 5.84 ± 0.1 | 1.29 ± 0.3 |
| *K. rheticus* LMG 22126 | GDG | glucose | ammonium | 0.26 ± 0.0 | 2.94 ± 0.1 | < 0.50 |
|  |  |  | urea | 0.22 ± 0.0 | 3.12 ± 0.1 | < 0.50 |
|  |  |  | peptone | 0.23 ± 0.0 | 3.13 ± 0.1 | 7.08 ± 0.8 |
|  |  | fructose | ammonium | 0.19 ± 0.0 | 3.55 ± 0.1 | 0.53 ± 0.2 |
|  |  |  | urea | 0.17 ± 0.0 | 3.90 ± 0.3 | < 0.50 |
|  |  |  | peptone | 0.17 ± 0.0 | 5.50 ± 0.1 | 4.33 ± 1.2 |
|  |  | sucrose | ammonium | 0.18 ± 0.0 | 3.47 ± 0.2 | < 0.50 |
|  |  |  | urea | 0.16 ± 0.0 | 3.91 ± 0.3 | < 0.50 |
|  |  |  | peptone | 0.13 ± 0.0 | 5.73 ± 0.1 | 6.29 ± 1.6 |
|  |  | maltose | ammonium | 0.18 ± 0.0 | 3.52 ± 0.2 | < 0.50 |
|  |  |  | urea | 0.16 ± 0.0 | 3.93 ± 0.3 | < 0.50 |
|  |  |  | peptone | 0.15 ± 0.0 | 5.96 ± 0.1 | 1.97 ± 1.9 |
|  |  | glycerol | ammonium | 0.21 ± 0.0 | 3.60 ± 0.1 | < 0.50 |
|  |  |  | urea | 0.16 ± 0.0 | 4.07 ± 0.3 | < 0.50 |
|  |  |  | peptone | 0.10 ± 0.0 | 5.95 ± 0.6 | 5.90 ± 0.4 |
| *K. rheticus* LMG 22126 | GDP | glucose | ammonium | 0.75 ± 0.1 | 2.45 ± 0.1 | 3.07 ± 1.5 |
|  |  |  | urea | 0.41 ± 0.3 | 2.56 ± 0.1 | 1.42 ± 0.4 |
|  |  |  | peptone | 0.15 ± 0.0 | 3.65 ± 0.1 | 3.19 ± 0.4 |
|  |  | fructose | ammonium | 0.38 ± 0.0 | 4.09 ± 0.4 | 2.97 ± 0.1 |
|  |  |  | urea | 0.24 ± 0.2 | 4.61 ± 0.6 | 1.98 ± 0.6 |
|  |  |  | peptone | 0.35 ± 0.2 | 5.88 ± 0.1 | 2.24 ± 0.2 |
|  |  | sucrose | ammonium | 0.32 ± 0.1 | 4.78 ± 1.1 | 1.72 ± 0.6 |
|  |  |  | urea | 0.21 ± 0.1 | 5.40 ± 1.4 | 1.78 ± 0.8 |
|  |  |  | peptone | 0.65 ± 0.5 | 4.61 ± 0.3 | 1.11 ± 1.1 |
|  |  | maltose | ammonium | 0.29 ± 0.0 | 4.52 ± 0.9 | 2.07 ± 1.2 |
|  |  |  | urea | 0.19 ± 0.1 | 5.08 ± 1.1 | 2.10 ± 0.8 |
|  |  |  | peptone | 0.06 ± 0.0 | 5.91 ± 0.1 | 1.77 ± 0.4 |
|  |  | glycerol | ammonium | 0.66 ± 0.2 | 3.76 ± 0.2 | 2.64 ± 0.2 |
|  |  |  | urea | 0.50 ± 0.2 | 4.92 ± 1.1 | 0.89 ± 0.1 |
|  |  |  | peptone | 0.05 ± 0.1 | 6.11 ± 0.1 | 1.66 ± 0.1 |
| *K. rheticus* LMG 22126 | GVP | glucose | ammonium | 0.49 ± 0.2 | 2.60 ± 0.1 | 6.71 ± 0.4 |
|  |  |  | urea | 0.35 ± 0.1 | 4.56 ± 2.6 | 5.80 ± 2.4 |
|  |  |  | peptone | 0.23 ± 0.1 | 3.16 ± 0.1 | 11.38 ± 0.6 |
|  |  | fructose | ammonium | 0.46 ± 0.1 | 3.15 ± 0.2 | 7.98 ± 1.3 |
|  |  |  | urea | 0.35 ± 0.1 | 4.91 ± 2.2 | 6.82 ± 3.9 |
|  |  |  | peptone | 0.15 ± 0.1 | 5.42 ± 0.1 | 9.28 ± 0.4 |
|  |  | sucrose | ammonium | 0.58 ± 0.1 | 3.36 ± 0.5 | 8.25 ± 1.3 |
|  |  |  | urea | 0.39 ± 0.1 | 5.26 ± 3.0 | 7.69 ± 2.2 |
|  |  |  | peptone | 0.05 ± 0.0 | 6.18 ± 0.1 | 7.75 ± 0.6 |
|  |  | maltose | ammonium | 0.49 ± 0.1 | 3.20 ± 0.3 | 7.01 ± 0.4 |
|  |  |  | urea | 0.41 ± 0.1 | 5.03 ± 2.5 | 7.36 ± 2.7 |
|  |  |  | peptone | 0.06 ± 0.0 | 6.07 ± 0.1 | 10.48 ± 0.3 |
|  |  | glycerol | ammonium | 0.56 ± 0.1 | 5.10 ± 2.5 | 1.06 ± 0.9 |
|  |  |  | urea | 0.39 ± 0.1 | 5.71 ± 3.5 | 3.16 ± 2.4 |
|  |  |  | peptone | 0.05 ± 0.0 | 5.33 ± 0.1 | 10.48 ± 0.3 |
| *K. swingsii* LMG 22125 | GSG | glucose | ammonium | 0.39 ± 0.0 | 2.82 ± 0.1 | 1.74 ± 0.8 |
|  |  |  | urea | 0.43 ± 0.1 | 2.94 ± 0.1 | 1.96 ± 0.6 |
|  |  |  | peptone | 0.14 ± 0.1 | 3.77 ± 0.1 | 2.14 ± 0.5 |
|  |  | fructose | ammonium | 0.33 ± 0.1 | 3.53 ± 0.3 | 1.30 ± 0.5 |
|  |  |  | urea | 0.40 ± 0.1 | 6.31 ± 1.2 | 1.29 ± 1.3 |
|  |  |  | peptone | 0.09 ± 0.1 | 6.15 ± 0.1 | 2.31 ± 0.5 |
|  |  | sucrose | ammonium | 0.21 ± 0.1 | 3.58 ± 0.2 | 1.63 ± 1.2 |
|  |  |  | urea | 0.21 ± 0.1 | 7.03 ± 0.5 | 2.41 ± 1.4 |
|  |  |  | peptone | 0.07 ± 0.1 | 6.99 ± 0.1 | 1.01 ± 0.9 |
|  |  | maltose | ammonium | 0.19 ± 0.1 | 3.84 ± 0.3 | 1.16 ± 1.1 |
|  |  |  | urea | 0.21 ± 0.1 | 7.00 ± 0.6 | 2.26 ± 0.7 |
|  |  |  | peptone | 0.06 ± 0.0 | 6.24 ± 0.1 | 1.57 ± 1.0 |
|  |  | glycerol | ammonium | 0.21 ± 0.1 | 3.45 ± 0.1 | 1.11 ± 0.4 |
|  |  |  | urea | 0.24 ± 0.1 | 6.53 ± 1.2 | 1.11 ± 0.4 |
|  |  |  | peptone | 0.02 ± 0.0 | 6.59 ± 0.1 | 2.14 ± 0.2 |
| *K. swingsii* LMG 22125 | GSP | glucose | ammonium | 0.50 ± 0.3 | 2.82 ± 0.1 | 1.63 ± 0.4 |
|  |  |  | urea | 0.66 ± 0.3 | 2.77 ± 0.1 | 2.00 ± 0. 7 |
|  |  |  | peptone | 0.08 ± 0.0 | 3.73 ± 0.1 | 0.77 ± 0.2 |
|  |  | fructose | ammonium | 0.29 ± 0.1 | 3.00 ± 0.2 | 1.60 ± 0.2 |
|  |  |  | urea | 0.48 ± 0.1 | 3.97 ± 0.3 | 2.18 ± 0.7 |
|  |  |  | peptone | 0.02 ± 0.0 | 5.81 ± 0.1 | 1.23 ± 0.1 |
|  |  | sucrose | ammonium | 0.22 ± 0.1 | 2.97 ± 0.1 | 2.42 ± 0.1 |
|  |  |  | urea | 0.29 ± 0.1 | 4.19 ± 0.7 | 2.72 ± 1.1 |
|  |  |  | peptone | 0.02 ± 0.0 | 7.07 ± 0.1 | 0.80 ± 0.1 |
|  |  | maltose | ammonium | 0.23 ± 0.1 | 3.03 ± 0.1 | 1.15 ± 0.1 |
|  |  |  | urea | 0.28 ± 0.1 | 4.13 ± 0.5 | 2.38 ± 0.7 |
|  |  |  | peptone | 0.19 ± 0.0 | 6.67 ± 0.1 | 0.70 ± 0.1 |
|  |  | glycerol | ammonium | 0.31 ± 0.1 | 3.02 ± 0.1 | 1.72 ± 0.6 |
|  |  |  | urea | 0.34 ± 0.1 | 4.01 ± 0.3 | 2.53 ± 0.9 |
|  |  |  | peptone | 0.06 ± 0.0 | 6.41 ± 0.1 | 0.92 ± 0.1 |
| *K. xylinus* LMG 1515 |  | glucose | ammonium | 0.16 ± 0.1 | 3.19 ± 0.1 | 0.75 ± 0.2 |
|  |  |  | urea | 0.15 ± 0.1 | 4.46 ± 1.3 | 1.38 ± 1.1 |
|  |  |  | peptone | 0.14 ± 0.1 | 3.67 ± 0.1 | 1.83 ± 0.2 |
|  |  | fructose | ammonium | 0.14 ± 0.0 | 3.36 ± 0.1 | < 0.50 |
|  |  |  | urea | 0.16 ± 0.1 | 4.70 ± 1.3 | 0.70 ± 0.7 |
|  |  |  | peptone | 0.10 ± 0.1 | 6.18 ± 0.2 | 2.80 ± 0.6 |
|  |  | sucrose | ammonium | 0.19 ± 0.0 | 3.43 ± 0.1 | < 0.50 |
|  |  |  | urea | 0.15 ± 0.1 | 5.30 ± 2.0 | 0.97 ± 0.8 |
|  |  |  | peptone | 0.12 ± 0.1 | 6.35 ± 0.4 | < 0.50 |
|  |  | maltose | ammonium | 0.12 ± 0.0 | 3.39 ± 0.1 | < 0.50 |
|  |  |  | urea | 0.18 ± 0.1 | 5.00 ± 1.6 | 0.75 ± 0.6 |
|  |  |  | peptone | 0.06 ± 0.0 | 6.54 ± 0.1 | < 0.50 |
|  |  | glycerol | ammonium | 0.14 ± 0.1 | 3.98 ± 0.1 | < 0.50 |
|  |  |  | urea | 0.32 ± 0.2 | 5.75 ± 2.1 | 0.39 ± 0.4 |
|  |  |  | peptone | 0.05 ± 0.0 | 6.61 ± 0.1 | < 0.50 |
| *K. xylinus* LMG 1518 |  | glucose | ammonium | 0.45 ± 0.1 | 2.73 ± 0.1 | < 0.50 |
|  |  |  | urea | 0.34 ± 0.1 | 2.72 ± 0.1 | 0.69 ± 0.1 |
|  |  |  | peptone | 0.73 ± 0.7 | 3.33 ± 0.1 | 4.67 ± 0.4 |
|  |  | fructose | ammonium | 0.79 ± 0.1 | 4.29 ± 0.6 | 0.53 ± 0.2 |
|  |  |  | urea | 0.34 ± 0.1 | 4.73 ± 0.8 | < 0.50 |
|  |  |  | peptone | 1.02 ± 0.1 | 5.16 ± 0.1 | 4.24 ± 0.1 |
|  |  | sucrose | ammonium | 0.33 ± 0.2 | 4.89 ± 1.4 | < 0.50 |
|  |  |  | urea | 0.50 ± 0.1 | 5.26 ± 1.2 | 0.92 ± 0.1 |
|  |  |  | peptone | 0.50 ± 0.3 | 4.00 ± 0.1 | 7.91 ± 0.6 |
|  |  | maltose | ammonium | 0.51 ± 0.1 | 4.79 ± 1.5 | < 0.50 |
|  |  |  | urea | 0.46 ± 0.1 | 5.26± 1.7 | 0.99 ± 0.8 |
|  |  |  | peptone | 0.57 ± 0.1 | 5.00 ± 0.1 | 1.70 ± 0.1 |
|  |  | glycerol | ammonium | 0.36 ± 0.1 | 3.99 ± 0.6 | 1.07 ± 0.1 |
|  |  |  | urea | 0.31 ± 0.1 | 4.02 ± 0.3 | 0.84 ± 0.5 |
|  |  |  | peptone | 0.69 ± 0.3 | 5.03 ± 0.1 | 3.82 ± 0.4 |
